# Genome-wide identification and functional prediction of novel and fungi-responsive lincRNAs in *Triticum aestivum*

**DOI:** 10.1186/s12864-016-2570-0

**Published:** 2016-03-15

**Authors:** Hong Zhang, Weiguo Hu, Jilei Hao, Shikai Lv, Changyou Wang, Wei Tong, Yajuan Wang, Yanzhen Wang, Xinlun Liu, Wanquan Ji

**Affiliations:** State Key Laboratory of Crop Stress Biology for Arid Areas, College of Agronomy, Northwest A&F University, Yangling, 712100 Shaanxi PR China; National Laboratory of Wheat Engineering, Institute of Wheat, Henan Academy of Agricultural Sciences, Zhengzhou, 450002 Henan China

**Keywords:** Wheat, Stripe rust, Powdery mildew, RNA-Seq, lincRNA, Sm-site, miRNA target

## Abstract

**Background:**

Stripe rust (*Puccinia striiformis* f. sp. *tritici*; *Pst*) and powdery mildew (*Blumeria graminis* f. sp. *tritici*; *Bgt*) are important diseases of wheat (*Triticum aestivum*) worldwide. Increasingly evidences suggest that long intergenic ncRNAs (lincRNAs) are developmentally regulated and play important roles in development and stress responses of plants. However, identification of lincRNAs in wheat is still limited comparing with functional gene expression.

**Results:**

The transcriptome of the hexaploid wheat line N9134 inoculated with the Chinese *Pst* race CYR31 and *Bgt* race E09 at 1, 2, and 3 days post-inoculation was recapitulated to detect the lincRNAs. Here, 283 differential expressed lincRNAs were identified from 58218 putative lincRNAs, which account for 31.2 % of transcriptome. Of which, 254 DE-LincRNAs responded to the *Bgt* stress, and 52 lincRNAs in *Pst*. Among them, 1328 SnRNP motifs (sm sites) were detected and showed RRU_4–11_RR sm site element and consensus RRU_1–9_VU_1–7_RR SnRNP motifs, where the total number of uridine was more than 3 but less than 11. Additionally, 101 DE-lincRNAs were predicted as targets of miRNA by psRNATarget, while 5 target mimics were identified using target mimicry search in TAPIR.

**Conclusions:**

Taken together, our findings indicate that the lincRNA of wheat responded to *Bgt* and *Pst* stress and played important roles in splicesome and inter-regulating with miRNA. The sm site of wheat showed a more complex construction than that in mammal and model plant. The mass sequence data generated in this study provide a cue for future functional and molecular research on wheat–fungus interactions.

**Electronic supplementary material:**

The online version of this article (doi:10.1186/s12864-016-2570-0) contains supplementary material, which is available to authorized users.

## Background

Recent studies have suggested that eukaryotic genomes encode a large number of functional transcripts of non-coding RNAs (ncRNAs), including housekeeping and regulatory RNAs [1–3]. The long ncRNA (lncRNA), one regulatory ncRNA, has been reported to be a vital component of eukaryotic gene regulation [4–7]. According to the length and general location, there are four types of long ncRNAs in plant, including long intron ncRNAs, promoter lncRNAs, long intergenic ncRNAs (lincRNAs) and natural antisense transcripts (lncNATs) [8, 9]. Determining the nature and possible biological functions of lncRNAs has been a rapidly developing field over the past decade [10]. A growing number of lincRNAs are known to be key regulators in higher eukaryotic organisms [11]. At present, human lincRNAs, lincRNAs in zebrafish, fruit fly and chicken have been well identified using large-scale sequencing. Comparing with the progress of long ncRNAs in animals, their study in plants starts relatively late. Yet, recent studies have identified numerous ncRNAs in plants with small genomes, including NATs in *Arabidopsis* and rice [12, 13], lincRNAs in maize, *Arabidopsis* and *Populus* [11, 14, 15], although mechanistic insights are still lacking. Additionally, 71 and 77 long npcRNA were predicted in wheat using Affymetrix Wheat Genome Array [16]. Hexaploid wheat (*Triticum aestivum*, AABBDD, 2*n* = 42) is one of the most widely grown and important food crops for human being with a large and complex genome. Use of the microarray analysis is often restricted by the known gene sequences arrayed on the chip, whereas RNA sequencing is not dependent on pre-existing databases of expressed genes and, therefore, provides an unbiased and more complete view of gene expression profiles [17], including lncRNA. However, few reports on genome-wide lncRNAs are available in bread wheat using high-throughput RNA sequencing.

Stripe rust (*Puccinia striiformis* f. sp. *tritici*; *Pst*) and powdery mildew (*Blumeria graminis* f. sp. *tritici*; *Bgt*) are important fungal diseases of wheat (*Triticum aestivum*) in many wheat-growing regions of the world, and as a result, significant crop damages occur in epidemic years [18–20]. In response to pathogen attack, plants have evolved sophisticated defence mechanisms to delay or arrest pathogen growth [21, 22]. Various gene transcript profilings have been used extensively to study wheat defenses against diseases [23–26]. Previously, we used large-scale sequencing to analysis the functional gene activation in wheat responding to stripe rust stress [27]. Here, inspired by long npcRNA function in wheat reponding to powdery mildew and heat stress [16] and our previously study on several lncRNAs’ roles in wheat responding to stripe rust pathogen infection [28], we aimed to identify lincRNA of wheat regulated in expression pattern after inoculation with *Pst* or *Bgt*, and to identify lincRNAs specific to the fungal stress response. We sequenced RNAs derived from leaf samples by RNA sequencing (RNA-seq) and captured the intergenic transcription units (TUs) encoding lincRNAs. Of which, differentially expressed lincRNAs were identified among treatment groups comparing with non-inoculated leaves as the control. To further validate and investigate the newly identified lincRNAs, we used qRT-PCR to profile several lincRNA expression in various time points of pathogene infected plants. We also predicted the funtion of lincRNA and profiled expression divergency of lincRNAs induced by stripe rust and powdery mildew.

## Methods

### Fungus and plant materials

The winter wheat line N9134, developed by Northwest A&F University, shows high resistance to *Pst* races CYR 29 and CYR 31 and is immune to all *Bgt* races in China. The *Pst* race CYR 31 was maintained by the College of Plant Protection of Northwest A&F University. The *Bgt* isolate E09 was maintained by the College of Agronomy. Seven-day-old seedlings were divided in two and inoculated with *Bgt* E09 or *Pst* race CYR 31 conidia, respectively. ‘Shaanyou 225’ and ‘Huixianhong’ were inoculated with E09 and CYR 31 to check inoculation effect. The inoculated leaves of N9134 were separately harvested at 0, 1, 2, and 3 days post-inoculation (dpi), frozen immediately in liquid nitrogen, and stored at −80 °C for RNA-Seq. The test was carried out with three biological replications.

### EST library construction and sequencing

Total RNA was extracted from samples of individual fungal-inoculated leaves at the specified time points using the TRIzol reagent (BioFlux, Hangzhou, China) method with a few modifications pertaining to DNase digestion and RNA purification. After RNA quality was checked as previously described [27], Oligo(dT)-magnetic beads were used to enrich the mRNA, which was then broken into fragments by fragmentation buffer. First-strand cDNA was prepared using a reverse transcription-PCR system (Promega, Madison, WI, USA) with random hexamers. Second-strand cDNA was synthesized using RNase H, DNA polymerase I and dNTPs. Poly(A) and adapter sequences were ligated to the ends of the repaired double-stranded cDNA after purification with a QiaQuick PCR kit. EST libraries were constructed by size selection and PCR amplification, and then sequenced with an Illumina HiSeq™TM 2000 platform by Biomarker Technology Co., Ltd (Beijing, China).

### Assembling RNA transcripts and identifying novel transcriptional units

After sequencing, paired-end reads were checked and scored with the standard of CycleQ20 level (a base quality greater than 20 and an error probability of 0.01). After cleaning low quality reads, all reliable readings were *de novo* assembled using the Trinity platform to reconstruct unigene library of wheat resistance line N9134 [29], and differential gene expression analysis was performed with the bioconductor package DESeq, version 3.2 [30]. After all annotated and pathway identified gene were removed, the long intergenic non-coding RNA were identified with rigorous criteria: (1) The transcript length must be more than 200 bps; (2) the transcript must contain no open reading frame (ORF) encoding more than 50 aa; (3) the TUs must not encode any transposable elements (TEs) and must not overlap with any encoding NATs; and (4) the transcript does not have intron (gap) comparing with wheat genome sequences in URGI (http://wheat-urgi.versailles.inra.fr/Seq-Repository). OrfPredictor was used to identify protein-coding regions in TUs, and to calculate the longest possible ORF of each strand. Then, the reads per kilobase of exon model per million of aligned readings (RPKM) values were used to examine the gene expression level distribution for each lincRNA in sample. Also, the correlation coefficients between repeats were calculated with statistical method.

### Bioinformatic analysis

Function annotation were conducted using the BlastX program against NCBI data bases and Gene Ontology (GO) and Kyoto Encyclopedia of Genes and Genomes (KEGG, http://www.genome.jp/kegg/genes.html) with E-Value ˂1E-5. The SnRNP motifs were predicted by RNA Analyzer with default parameter [31]. The plant small RNA target analysis server, psRNATarget, was used to predict the miRNA targets of lincRNA with stringent cut-off threshold 3.0 in miRBase database [32].

### Quantitative Real-Time PCR analysis

SYBR green Premix Ex TaqTM II quantitative PCR system was used for qPCR analysis (TaKaRa, Dalian, China). All experiments involving Q-PCR were performed on a 7300 Real-Time PCR System (Applied Biosystems, Forest City, CA, USA) using primers described in Additional file 1: Table S4 online. The RNA samples used as templates for RNA-Seq were the same as those used for qPCR. Additionaly, the RNA were extracted from additional stages of innoculated wheat leaves at 0.5, 1.5, 4, and 5 dpi, and used for analyzing the expression pattern of lincRNA together. Sample cycle threshold (Ct) values were determined and standardized relative to the endogenous control tubulin gene (GenBank: U76558), and the 2^–∆∆CT^ method was used to calculate the relative changes of gene expression in fungi-inoculated plants vs. mock-inoculated plants. PCR was conducted according to standard protocol in triplicate [28].

## Results

Previously, Gene expression profile for the response to the stripe rust and powdery mildew pathogens in wheat were analyzed after cDNA libraries were constructed from leaves inoculated with Pst or Bgt at 0, 1, 2 and 3 dpi with three biological replicates, and sequenced using the Illumina HiSeq™ 2000 platform. The correlation coefficient values ranged from 0.930 to 0.994 as shown in previous report [27]. Here to identify novel and fungi-responsive lincRNAs, these TUs were analysed further using computational and experimental methods. After these reads were *de novo* assembled using the Trinity platform software, four filter processes were applied to distinguish lincRNAs and alignmented with wheat genome sequences for transcript units.

### Identification of lincRNA candidates responding to fungi stress

**Fig. 1:**
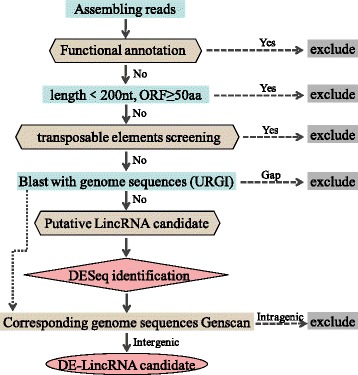
Pipeline of data from RNA-Seq to DE-lincRNA candidates responding to stripe rust and powdery mildew. Sequence reads were assembled using the Trinity platform and all unigenes were annotated in NCBI, COG, GO and KEGG. Unknown transcripts were filtered using thresholds of ORF length and nucleotide length. After filtering, transcripts were further alignmented with genome sequences downloaded from URGI, and then those genes that have not gap were reserved as putative lincRNA. The differential expression (DE)-lincRNAs were identified on the condition of fold change ≥2 and the false discovery rate (FDR) at 1.0 %. Then, DE-lincRNA were further verified though analysis of corresponding genome sequences by Genscan

Since RNA-seq data and *de novo* assembled unigenes were used to analyze these ncRNAs, the NATs are could not be identified due to missing transcribe directional control. Additionally, some of ncRNA may also be related to other types of transcripts, such as truncated mRNAs, by-products of protein-coding genes, expressed repeats, or other ncRNAs. Such transcripts may confound the analysis of bona fide lincRNAs. Therefore, to facilitate further investigation of lincRNAs, a pipeline for the identification of lincRNAs was constructed with the aforementioned criteria (Fig. 1). This referenced Liu et al. [15] and Ariel et al. [8] described criteria, but provide a more strict definition for lincRNAs. Taking unintegrated unigene into consideration, a total of 186,632 unigenes were found in the seven libraries. Of which, 96,960 unigenes were not annotated after Blast searches of the GenBank Nr, SwissProt, KEGG, COG and GO databases. On the condition of the length of unigenes were more than 200 nucleotide base pairs (bps), the putative protein-coding RNAs were then filtered using a maximum possible ORF length of 50 amino acids (AA). Furthermore, these candidate lincRNAs were selected using BlastN against wheat genome sequences of URGI. After these two steps, a totals of 58,218 novel intergenic transcriptional units found in seven libraries were selected as putative lincRNAs. Setting fold change ≥2 and the false discovery rate (FDR) at 1.0 %, statistical analysis with DESeq identified 283 lincRNA loci as differentially expressed among the six treatment groups compared with non-inoculated leaves as the control. Using inoculated leaf samples, expression of 254 DE-LincRNAs were detected in the *Bgt* test, while 52 lincRNAs were differentially expressed in *Pst* test. Of which, 23 DE-lincRNAs overlapped between the two infection treatments.

**Fig. 2:**
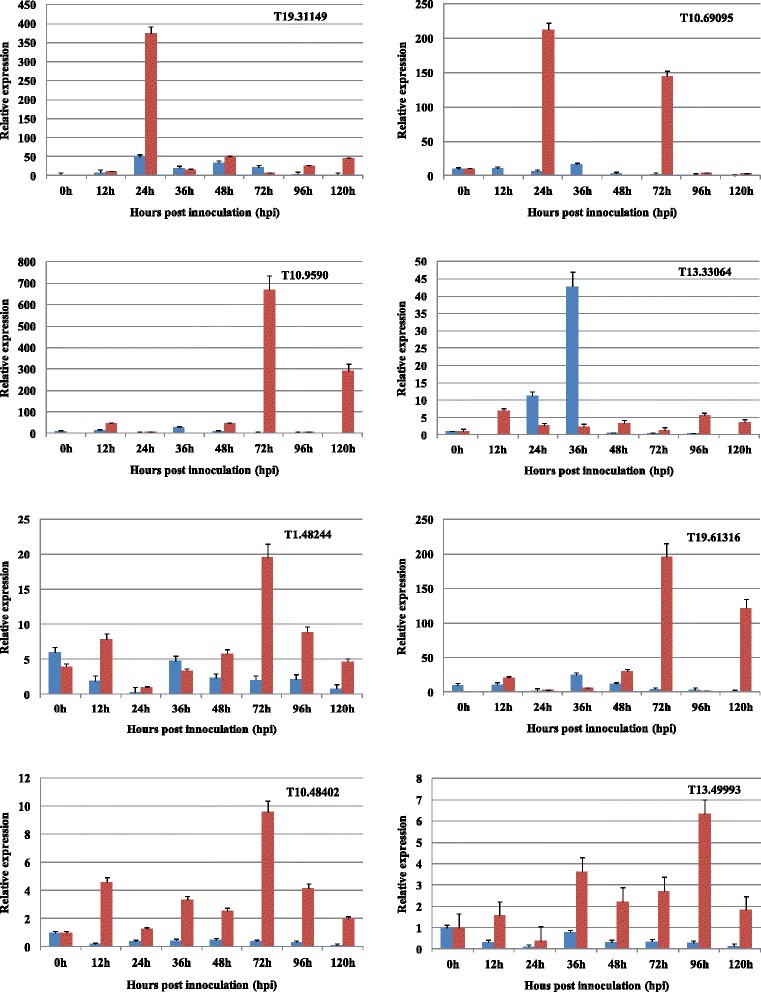
Expression patterns of selected DE-lncRNAs in N9134 induction by stripe rust and powdery mildew pathogen at 0, 12, 24, 36, 48, 72, 96 and 120 hpi. Gene expression levels were assessed by qRT-PCR. Data were normalized to the α-tublin expression level. The mean expression value was calculated from three independent replicates. Red bar means the gene expression in wheat infected by *Pst*, while blue bar represents *Bgt* pathogen stress

To evaluate the reliability of DE-lincRNA, quantitative real-time PCR (qPCR) was performed on nine random selected LincRNAs using RNA samples. These genes were selected to represent a wide range of expression levels and patterns under fungal infection. These gene expression patterns in response to fungal stress suggested their participation in pathogen-defense responses as detected in RNA-Seq and shown to be differentially expressed in wheat after *Pst* and *Bgt* inoculation (Fig. 2). The statstics analysis showed that infection by *Bgt* affected 98, 112 and 144 DE-LincRNA at 1, 2 and 3 dpi respectively, while 30, 18 and 14 DE-LincRNAs were detected at the corresponding time points in *Pst* stress. Of which, 25–38 % lincRNA are shared by different time points in *Bgt* stress, and a few lincRNAs overlapped at any two timepionts could be seen in N9134 responding to *Pst* stress. This menas that the most of lincRNAs vary with the stages of fungal-infected wheat.

### Length and scaffold distribution of lincRNAs in wheat genome

Based on the above results, 58,218 putative lincRNAs were selected for further analysis. Because of the interest in the chromosome set distribution of differential expression genes, we filtered all 58,218 putative lincRNA with genome sequences on condition that Identify ˃98 %. The result showed that 23,358 unigenes could be perfect mapped into wheat genome with E-Value ˂1E-10 and Identify ˃98 %, of which, 9328 unigenes were mapped to chromosome set A, 9711 to chromosome B and 9552 to chromosome D. The number of genes that could be mapped to both A and B reaches to 1917, which is less than 2124 genes mapped to A and D, and 2049 to B and D. Then the mapped genes were further divided into detailed chromosome and listed in Table 1.

**Table 1:** The distribution of lincRNAs mapping into draft wheat genome

Chromosome Set/Arm	Homologous group						
	1	2	3	4	5	6	7
AL	840	1074	959	1123	1116	690	739
AS	584	822	715	653	497	549	781
BL	840	1225	1828	767	1467	1031	974
BS	573	800		778	442	928	623
DL	925	1407	1133	1125	1035	690	732
DS	404	734	676	477	456	549	827

**Fig. 3:**
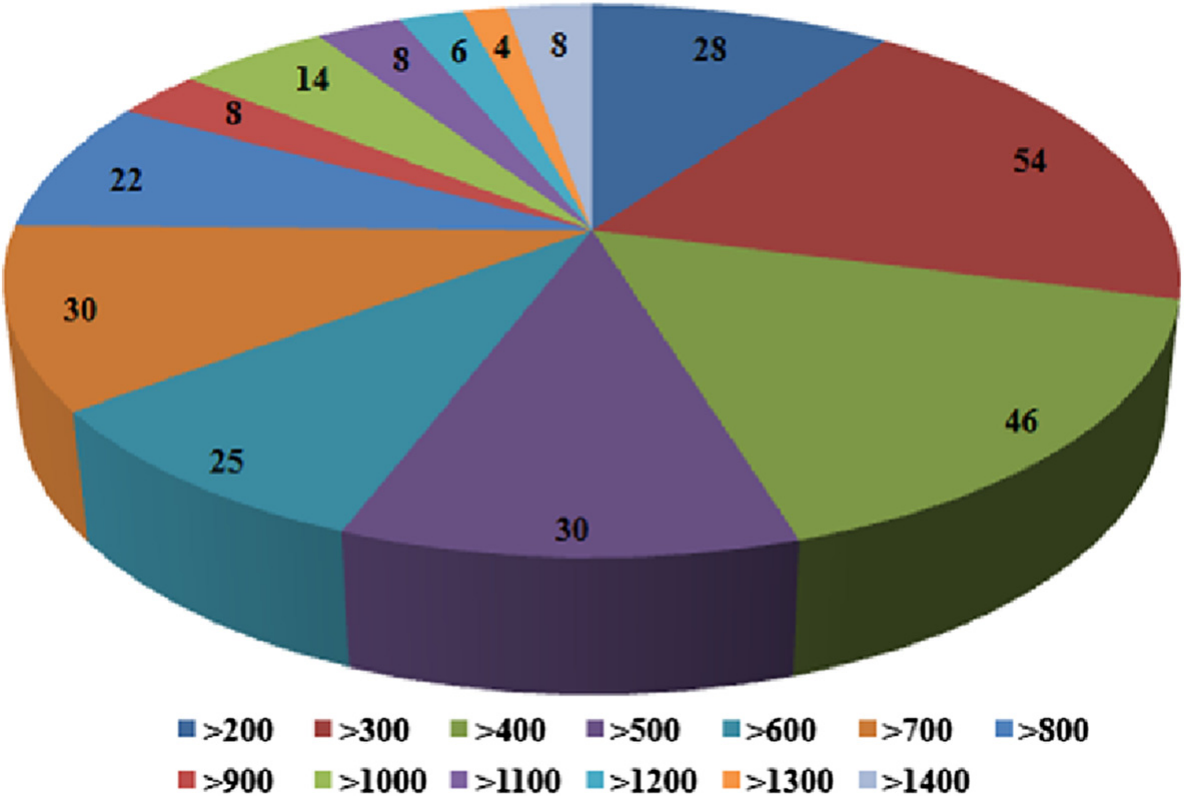
The length distribution of 283 DE-lincRNAs in fungal infected leaves comparing with non-inoculated. The numbers of DE-lincRNA in different length range were marked on the pie plots. The different color indicated different length range were listed in the bottom panel

The mapping result showed that DE lincRNA dysregulated by fungi come from all chromosomes of wheat, which indicated that plant resisting pathogen is a colossally complex system and that is similarly with functional DE genes. Also this suggests that we should concentrate interest on partial genes, such as 1B and 5B, where the *Pst* and *Bgt* resistance genes were located respectively. The length distribution of these DE-lincRNAs loci ranged from 212 bp to 3151 bp, yet more than 80 % ranged from 200 bp to 800 bp (Fig. 3). The average length was 635 bps, while the most abundant length was 300–500 bp.

### Sm-sites diversity of lincRNAs in wheat reponding to fungi stress

Since a role of lncRNAs in mammalian cells is to bind and sequester several serine/arginine (SR) splicing factors, leading to altered pattern of alternative splicing for a set of pre-mRNAs [5], we analyzed the SnRNP motifs characterization of DE-lincRNAs. The results of RNA Analyzer [31] detected 1328 SnRNP motifs (including sm sites), as well as 28 element 2a/2b from 246 differential expressed lincRNAs (both sense and antisense strand) in N9134 responding to powdery mildew and stripe rust pathogen stress (Table 2 and Additional file 1: Table S1). Furthermore, the predicted SnRNP motifs were manul scanning with Bioedit software. The statistics analysis indicated that several DE-lincRNA contains over 10 SnRNP motifs, such as T13.34604, T16.15844 and T19.56184. Because those elements are indications for processing protein binding motifs, we infer that those lincRNA play critical role in wheat responding to fungal infection. These motifs were further classed into 47 putative Sm-site and 407 SnRNA oligonucleotide. The sm site element of U1, U2, U4/6, U5 spliceosomal snRNA is characterized with consensus PuAU_3–6_GPu. However, the statistical result showed that the sm site motifs contained 4 to 11 uridine in wheat, namely RRU_4–11_RR construct. Among Sm-site motifs, the consensus of AAUUUUGA is presented with the highest frequency and followed by AAUUUUAA and AGUUUUAG, although the DE-lincRNAs harboring it are specific induced by stripe rust and powdery mildew. The SnRNP motifs showed more complex consensus RRU_1–9_VU_1–7_RR, where the total number of uridine is more than 3 but less than 11 (where R can be adenine or guanine and V indicates any nucleoside but not uridine). These mean that one more complicated SnRNP and ncRNP spliceosome and more diversified mRNA resulting in flexibility in protein sequences. This also hints that lincRNAs play an important role in alternative splicing.

**Table 2:** Putative sm-sites of DE-lincRNAs in wheat responding to stripe rust and powdery mildew

Sequences	Fungi	Sequences	Fungi	Sequences	Fungi
gauuuuga	+	aguuuugg	P	aauuuuuag	P
aauuuugg	P	gguuuuaa	−	aguuuuuga	P
*aguuuuag	P	gauuuuaa	+	gauuuugg	+
*aauuuuaa	−	gguuuuuuga	P	gauuuuuuag	P
gguuuuuga	P	gauuuuag	−	aauuuuuuuga	P
aauuuuag	±	gauuuuugg	P	aguuuuga	−
aguuuuaa	−	aguuuuuuuuuuag	P	aauuuuuga	P
gauuuuuga	P	gguuuuuag	P	gguuuugg	P
gguuuuuuuuuuugg	+	gauuuuuuuuuaa	P	gauuuuuugg	P
gauuuuuaa	S	aauuuuuugg	P	gauuuuuag	P
*aauuuuga	±	aauuuuuuugg	−	gguuuuag	−
gguuuuga	−	aguuuuugg	P	aauuuuuuuuag	S
aguuuuuag	±	aguuuuuuuuuaa	P	aguuuuuaa	P
aauuuuugg	−	aauuuuuaa	P	gauuuuuuuaa	P
gguuuuuuaa	P	aguuuuuugg	P	gguuuuuaa	P
aauuuuuuaa	P	gauuuuuuaa	P		

The sm-site were predicted with RNA Analyzer online. P means that the motif harbored in the DE-lincRNA induced by powdery mildew. S means that the motif was harbored by the DE-lincRNA induced by stripe rust. “+” means that the DE-lincRNA carrying the corresponding motif was induced by both fungi. “−”means the motif was carried by several DE-lincRNAs but the latter were specific induced by stripe rust and powdery mildew respectively. The high frequent motifs were marked with star

### Identification of lincRNAs as Putative targets and target mimic of miRNAs

**Fig. 4:**
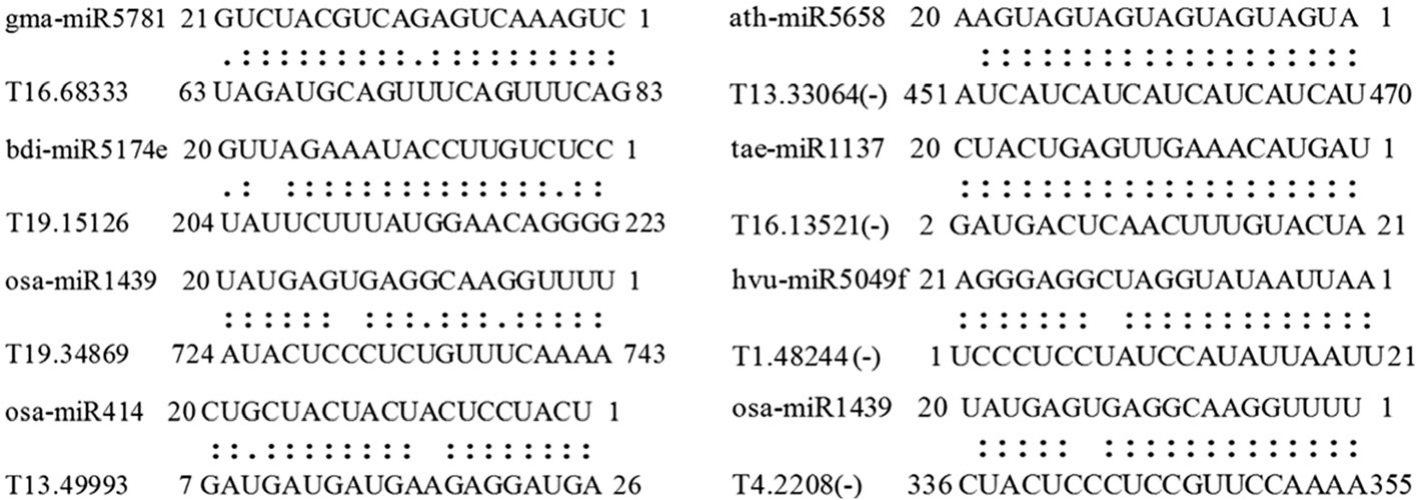
Putative targets of lincRNAs. Eight lincRNAs as miRNA targets are shown. (–) represents antisense strand of lincRNA. The name of target lincRNA and the matched miRNA were given in the left of each sequence

Considering that the relationship between miRNA and lncRNA is also an important issue. This means the miRNAs may play roles in promoting the degeneration of lincRNAs, so we screened the miRNA target sites in DE-lincRNAs. Using psRNATarget [32], a total of 101 lincRNAs were predicted as target of miRNA. Of which, 59 lincRNAs were targeted at sense strand, while 63 at the antisense strand (Additional file 1: Table S2). Figure 4 showed several putative miRNA target lincRNAs on the sense strand and antisense strand. The total of 39 lincRNA were targeted by miRNA for inhibiting translation activation. Of which, three lincRNA were identified as putative targets of miRNAs on both antisense and sense strand. Among these miRNA, six related miRNAs were detected by Feng in our laboratory, using small RNA deep sequencing. Gene expression proved that they are implicated in Xingzi 9104 responding to stripe rust pathogene CYR 32, such as miR156, miR160, miR164, miR167, miR393, miR398, miR829, etc [33], while Xin et al substantiated that some of them are involved in powdery mildew stress [34]. Intriguingly, apart from different miRNAs target the same lincRNA, some lincRNA have multiple target sites. For example, T13. 49993 showed double repeated target sites of ath-miR414 with cleavage inhibition, while contains one target site of osa-miR414 functioned on translation inhibition as shown on Fig. 4. Additionally, considering large amount of functional encoding genes were implicated in wheat responding to fungi stress, the functional genes target of several key miRNA were predicted with an expected threshold value less than 2.0 and listed in Additional file 1: Table S3. The results showed that ath-miR414 regulated 25 functional genes; 47 functional genes regulated by ath-miR5658, 5 by tae-miR1137a, and so on. This hints that lincRNA could competitive interplay with functional genes via miRNA regulation.

**Fig. 5:**
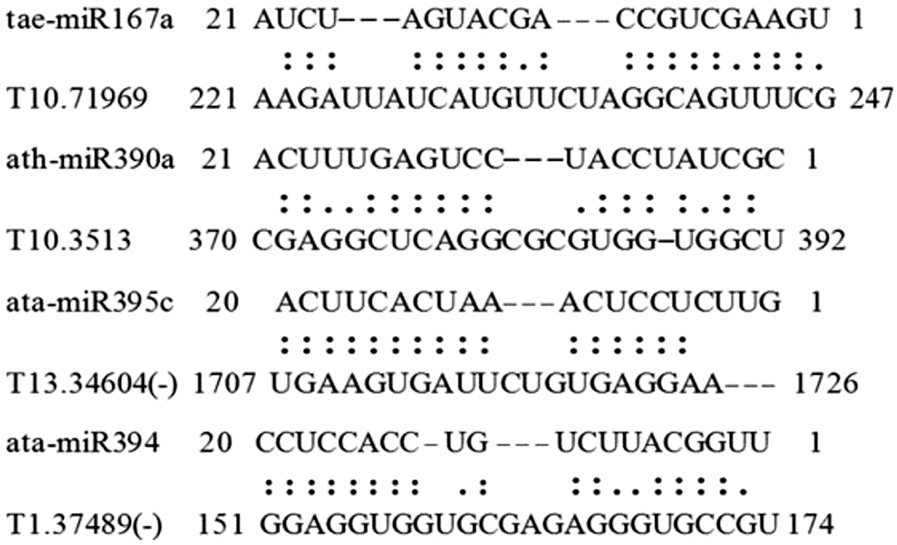
Putative targets mimics of lincRNAs. Four lincRNAs as target mimics of miRNAs are shown. (–): antisense strand of lincRNA. The name of miRNA and the target mimic lincRNAs were listed in the left. The Start and End site were presented at both ends of each sequence

In addition, inspired by that functional eTMs may be composed mainly of lncRNAs [35], we identified intergenic or noncoding gene-originated endogenous microRNA target mimic (eTM) for conserved miRNAs from *Arabidopsis thaliana*, wheat (*T. aestivum*) and goat grass (*Aegilops tauschii*). Here, using target mimicry search in TAPIR, 5 target mimics were identified on the sense and antisense strand of these DE-lincRNAs responding to fungi (http://bioinformatics.psb.ugent.be/webtools/tapir/). They could bind to tae-miR167a, ath-miR390a, ata-miR156d-3p, ata-miR160a-3p, ath-miR394a, ata-miR395c-5p and ath-miR399b (Fig. 5) with a three-nucleotide bulge between the 5′ end 10th to 11th positions of miRNAs. Among of these miRNA, ata-miR395c-5p was pairing with lincRNA T13.34604 as target mimicry, which is the target of osa-miR5834, stu-miR8007b-5p and mtr-miR2634. This means that the DE-lincRNA, T13.34604 containing 12 SnRNP motif and one sm site as aforementioned, plays double roles in the regulatory mechanism, i.e., it inhibits ata-miR395c function but is regulated by osa-miR5834 homologue. Moreover, miRNA394 perfects pairing with T4.41043, a F-box protein of wheat in a previously study [27]. Here, the results showed the lincRNA, T1.37489, could inhibit the degradation activation of miRNA394 targeting F-box functional gene specifically. This suggested that lincRNA 37489 regulated F-box protein gene via regulating miRNA394 cleavage function.

### Co-expression of miRNA targeted functional genes and lincRNAs

**Fig. 6:**
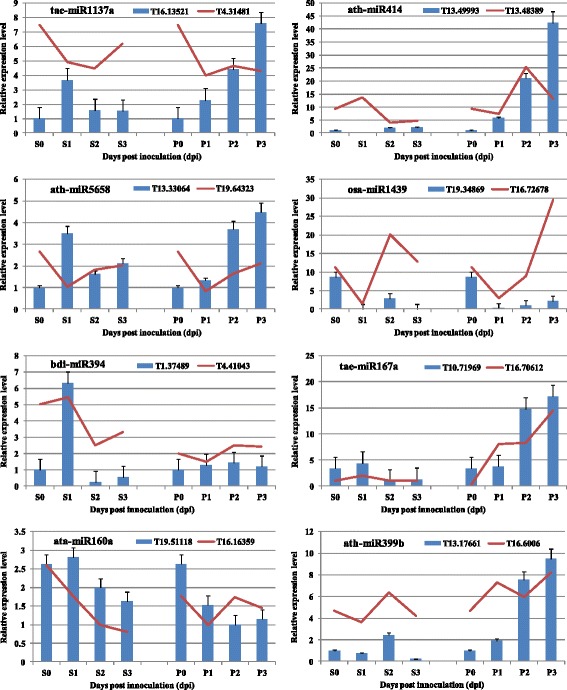
Co-expression patterns of selected miRNA targeted lncRNAs and functional genes in N9134 induction by stripe rust and powdery mildew pathogen. Gene expression levels were assessed by transcript accumulation analysis. The mean expression value was calculated from three independent replicates. Bar charts means the gene expression of lincRNA in wheat infected by *Pst* and *Bgt*, while line charts represent transcription expression of functional genes. The number 0 1, 2 and 3 mean that N9134 was infected at 0, 1, 2 and 3 dpi, respectively. P represents powdery mildew E09 inoculation condition; S represents stripe rust pathogen CYR 31 inoculation. The name of miRNA and corresponding targeted gene were listed in the top of each panel. The lincRNAs in top four charts were targeted by miRNAs for cleavage inhibition, while the bottom four were target mimic of miRNAs

LncRNAs may regulate protein-coding gene expression level as competing endogenous RNAs to regulate miRNA levels [7]. Here to verify aforementioned analysis, we analyzed the expression correlation between miRNAs targeted lincRNAs and functional genes using selected eight pair genes, which targeted by tae-miR1137a, ath-miR414, ath-miR5658, osa-miR1439, bdi-miR394, tae-miR167a, ata-miR160a-3p and ath-miR399b. Among of them, the first four miRNAs were predicted to target lincRNA for cleavage, while the last four were targeted by lincRNA as target mimics. The relative expression of lincRNAs and corresponding functional genes in the leaves of N9134 infected with CYR31 and E09 are shown in Fig. 6. Following inoculation with *Pst* or *Bgt*, both lincRNAs (T16.13521, T13.49993, T13.33064, T19.34869, T1.37489, T10.71969, T19.51118, T13.17661) and functional gene transcripts (T4.31481, T13.48389, T19.64323, T16.72678, T4.41043, T16.70612, T16.16359, T16.6006) showed dysregulated expression. Intriguingly, the expression profiles of most lincRNA target mimic transcripts of miRNA were consistant with the expression of functional genes targeted by the same miRNA. However, the transcription level correlation between miRNAs targeted lincRNAs and functional genes is more complex, although some lincRNA target performed the semblable fluctuation in inoculated plants vs. protein-coding gene (Fig. 6). For example, the expression of miRNA414 targeted lincRNA T13.49993 increased stably at 1, 2 and 3 dpi after innoculation with *Bgt*, whlie the targeted functional gene T13.48389 was induced 2.7-fold at 2 dpi but followed by a steep decrease at 3 dpi. The transcripts peak of miRNA5658 targeted lincRNA T13.33064 was detected at 1 dpi after inoculation with *Pst*, whereas the expression of protein-coding gene T19.64323 reach to the lowest value at this time point. This findings indicate that lincRNAs were involved in funtional gene regulation via miRNAs but the interaction network seems to be very complex in wheat.

## Discussion

Literatures of lncRNAs in various biological processes of mammal and plants have bursting emerged in recent years [5, 8, 36]. These efforts have identified a myriad of molecular functions for lncRNAs. The lncRNAs are perceived to play central roles of gene regulation in responding to biotic and abiotic stress and may form the basis of an inter-gene communication system, including RNAi and modifying chromatin structure [1, 7, 37] but not the ‘junk’. The complex molecular mechanisms of lncRNAs is beyond the role that lncRNAs play a role as primary transcripts to produce short RNAs [4, 38, 39]. Previously, we isolated three lincRNA and one lncNAT cDNAs, which are differentially expressed in wheat after *Pst* inoculation [28]. Here, using the RNA sequencing (RNA-Seq), we identified 58,218 lincRNAs from seedlings of wheat at the three-leaf stage and predicted the function of 283 DE-lincRNA implicated in the interaction of wheat with *Pst* and *Bgt*. As new lncRNAs are being discovered at a rapid pace in mammal and model plant, the molecular mechanisms of lincRNA would be enriched and diversified in wheat because of the demonstration effect given in this text, and further lay the foundation for investigating the functions of lncRNAs and the mechanism of wheat defending *Pst* and *Bgt*.

Small nuclear ribonucleic proteins (snRNPs) are RNA-protein complexes that combine with pre-mRNA and various other proteins to form a spliceosome, manipulating splicing of pre-mRNA [40]. The two essential components of snRNPs are Sm protein molecules and small nuclear RNA (snRNA). The snRNA gives specificity to individual introns by “recognizing” the sequences of critical splicing signals at the 5′ and 3′ ends and branch site of introns [41, 42]. The Sm class of snRNAs is comprised of U1, U2, U4, U4atac, U5, U7, U11 and U12, whereas the lsm class is made up of U6 and U6atac [43]. Their nomenclature derives from their high uridine with the Sm site consensus 5′-RAU_3–6_GR-3′. For example, the oligonucleotide 5′-AAUUUUUGA-3′contacts to human SmB/B’ [44]; the loose consensus 5′-AAYYrY(U)R-3′ (Sm site of U2 snRNA) was detected in trypanosomes [45]. Recent study highlight a regulatory strategy for transcriptional control via specific RNA-RNA interaction between U1 snRNA and exon-intron circRNAs [46], as well as the circRNAs are most probably noncoding [47]. Although the length of ncRNA is different from snRNA (about 150 nucleotides averagely), the similar motifs of sm site exited in lincRNA. Additionally, the ncRNA activity is typically driven by base pairing and often involves several partner proteins [48], named as the non-coding ribonucleoprotein (ncRNP) [43]. Comparing the Sm site sequences in wheat with those in mammals and trypanosomes snRNAs revealed a striking difference. the number of pyrimidine stretch in Sm sites (RRU_4–11_RR) reach to 11, while the proximate flanked positions are any of purine but not univocal adenine or guanine. In contrast, a losser consensus 5′- RRU_1–9_VU_1–7_RR -3′ can be derived for the other snRNP motifs, an unusual purine or cytosine position interrupts the central pyrimidine stretch in the middle. Taken together, this hints lincRNA may play a critical role in pre-mRNA splicing, but the function should be more complex. U2-type introns have GT-AG at their 5′ and 3′ ends while U12-type introns have AT-AC splice sites. Intriguingly, we firstly found an unexpected and striking alternative splicing of *TaNAC1* in wheat (Zhang unpublished). This substantiated the inference as an experimental evidence, although the interaction should be further dissected.

LincRNAs are similar in nature to mRNA in that miRNAs can bind lincRNAs and trigger degradation [49]. The inter-regulation between miRNA and lincRNA is a novel component of miRNA regulation, including lincRNA repression and miRNA target mimic. Target mimicry is an identified miRNA regulation mechanism first reported in *Arabidopsis* [50]. Recently, since the increasing data on ncRNA were explored, miRNA target lincRNA mimics have yet been identified in *Arabidopsis* and *Populus* [11, 35]. Comparing with the previous report on long npcRNA using the wheat Affymetrix Gene Chip [16], here, much more lncRNA were observed. The predictions were performed using 283 DE-lincRNAs and 101 potential miRNA targets and 5 target mimics were firstly identified in wheat responding to *Pst* and *Bgt* stress. The ratio of target reach to 35.7 % of differential expressed lincRNA. It is worth noting that previously experiment showed that miRNAs and their corresponding targets could construct a very complex interactive regulation network, because some miRNAs could even target nearly ten different target genes, whlie some gene could be regulated by several miRNAs [33]. This is similar with the finding of lincRNA and protein coding gene interacting with miRNA here. Taken these findings together, we could concluded that LincRNAs have more complicated roles in responding to biotic and abiotic stresses in plants, in addition to the action of miRNA precursors. With the recent demonstration for lncRNAs, the activation of their transcription is sufficient for function, it becomes clear that there could be a number of lncRNAs acting in a similar way. If the above described findings are used as guidelines, many new lncRNAs regulating genes will be discovered, which will be helpful to understanding plant resistance to fungi.

## Conclusions

Infection by *Bgt* and *Pst* triggered robust alteration in gene expression of lincRNAs in *T. aestivum*. These DE-lincRNA showed RRU_4–11_RR sm site element and consensus RRU_1–9_VU_1–7_RR SnRNP motifs. This displayed a more complex sm site construction in wheat than that in mammal and model plant. Half DE-lincRNAs were predicted as targets of miRNA, while several lincRNAs are target mimics of miRNA. Our findings indicate that the lincRNA of wheat responded to *Bgt* and *Pst* stress and played important roles in splicesome and inter-regulating with miRNA.

## Availability of data and materials

The dataset supporting the conclusions of this article is available in its additional file and the NCBI repository http://www.ncbi.nlm.nih.gov/bioproject/?term=%20PRJNA243835.

## Additional file

Additional file 1: Table S1. SnRNP motif of wheat lincRNAs detected by RNA Analyzer. **Table S2.** The list of DE-LincRNAs as putative targets of miRNAs and its sequences. **Table S3.** Key miRNA target of functional gene at sense and antisense strand. **Table S4.** PCR primers used for Q-PCR amplification of DE-lincRNA. (PDF 857 kb)

## Abbreviations

circRNA: circular RNA
lincRNA: long intergenic ncRNA
lncNAT: natural antisense transcript
lncRNA: long non-coding RNA
miRNA: microRNA
ncRNA: non-coding RNA
npcRNA: non-protein coding RNA
Sm site: conserved nucleotide sequence of snRNA binding to Sm proteins
snRNA: small nuclear RNA
snRNP: small nuclear ribonucleoprotein complexes

## References

[1] Charon C, Moreno AB, Bardou F, Crespi M (2010). Non-protein-coding RNAs and their interacting RNA-binding proteins in the plant cell nucleus. Mol Plant.

[2] Huttenhofer A, Schattner P, Polacek N (2005). Non-coding RNAs: hope or hype?. Trends Genet.

[3] Brosnan CA, Voinnet O (2009). The long and the short of noncoding RNAs. Curr Opin Cell Biol.

[4] Kim ED, Sung S (2012). Long noncoding RNA: unveiling hidden layer of gene regulatory networks. Trends Plant Sci.

[5] Wang KC, Chang HY (2011). Molecular mechanisms of long noncoding RNAs. Mol Cell.

[6] Wilusz JE, Sunwoo H, Spector DL (2009). Long noncoding RNAs: functional surprises from the RNA world. Genes Dev.

[7] Kornienko AE, Guenzl PM, Barlow DP, Pauler FM (2013). Gene regulation by the act of long non-coding RNA transcription. BMC Biol.

[8] Ariel F, Romero-Barrios N, Jegu T, Benhamed M, Crespi M (2015). Battles and hijacks: noncoding transcription in plants. Trends Plant Sci.

[9] St Laurent G, Wahlestedt C, Kapranov P (2015). The Landscape of long noncoding RNA classification. Trends Genet.

[10] Atkinson SR, Marguerat S, Bahler J (2012). Exploring long non-coding RNAs through sequencing. Semin Cell Dev Biol.

[11] Shuai P, Liang D, Tang S, Zhang Z, Ye CY, Su Y, Xia X, Yin W (2014). Genome-wide identification and functional prediction of novel and drought-responsive lincRNAs in Populus trichocarpa. J Exp Bot.

[12] Li L, Wang X, Sasidharan R, Stolc V, Deng W, He H, Korbel J, Chen X, Tongprasit W, Ronald P (2007). Global identification and characterization of transcriptionally active regions in the rice genome. PLoS One.

[13] Wang H, Chung PJ, Liu J, Jang IC, Kean MJ, Xu J, Chua NH (2014). Genome-wide identification of long noncoding natural antisense transcripts and their responses to light in Arabidopsis. Genome Res.

[14] Boerner S, McGinnis KM (2012). Computational identification and functional predictions of long noncoding RNA in Zea mays. PLoS One.

[15] Liu J, Jung C, Xu J, Wang H, Deng S, Bernad L, Arenas-Huertero C, Chua NH (2012). Genome-wide analysis uncovers regulation of long intergenic noncoding RNAs in Arabidopsis. Plant Cell.

[16] Xin M, Wang Y, Yao Y, Song N, Hu Z, Qin D, Xie C, Peng H, Ni Z, Sun Q (2011). Identification and characterization of wheat long non-protein coding RNAs responsive to powdery mildew infection and heat stress by using microarray analysis and SBS sequencing. BMC Plant Biol.

[17] Ozsolak F, Milos PM (2011). RNA sequencing: advances, challenges and opportunities. Nat Rev Genet.

[18] Chen W, Wellings C, Chen X, Kang Z, Liu T (2014). Wheat stripe (yellow) rust caused by Puccinia striiformis f. sp. tritici. Mol Plant Pathol.

[19] Li Z, Lan C, He Z, Singh RP, Rosewarne GM, Chen X, Xia X (2014). Overview and application of QTL for adult plant resistance to leaf rust and powdery mildew in wheat. Crop Sci.

[20] Kang ZS, Zhao J, Han DJ, Zhang HC, Wang XJ, Wang CF, Han QM, Guo J, Huang LL (2010). Status of wheat rust research and control in China. BGRI 2010.

[21] Staal J, Dixelius C. Plant Innate Immunity. In: Encyclopedia of Life Sciences (ELS). Chichester: John Wiley & Sons, Ltd; 2009. DOI:10.1002/9780470015902.a0020114.

[22] Jones JD, Dangl JL (2006). The plant immune system. Nature.

[23] Yu X, Wang X, Wang C, Chen X, Qu Z, Yu X, Han Q, Zhao J, Guo J, Huang L (2010). Wheat defense genes in fungal (Puccinia striiformis) infection. Funct Integr Genomics.

[24] Bhuiyan NH, Selvaraj G, Wei Y, King J (2009). Gene expression profiling and silencing reveal that monolignol biosynthesis plays a critical role in penetration defence in wheat against powdery mildew invasion. J Exp Bot.

[25] Coram TE, Settles ML, Chen X (2009). Large-scale analysis of antisense transcription in wheat using the Affymetrix GeneChip Wheat Genome Array. BMC Genomics.

[26] Wang X, Liu W, Chen X, Tang C, Dong Y, Ma J, Huang X, Wei G, Han Q, Huang L (2010). Differential gene expression in incompatible interaction between wheat and stripe rust fungus revealed by cDNA-AFLP and comparison to compatible interaction. BMC plant biology.

[27] Zhang H, Yang Y, Wang C, Liu M, Li H, Fu Y, Wang Y, Nie Y, Liu X, Ji W (2014). Large-scale transcriptome comparison reveals distinct gene activations in wheat responding to stripe rust and powdery mildew. BMC Genomics.

[28] Zhang H, Chen X, Wang C, Xu Z, Wang Y, Liu X, Kang Z, Ji W (2013). Long non-coding genes implicated in response to stripe rust pathogen stress in wheat (Triticum aestivum L.). Mol Biol Rep.

[29] Haas BJ, Papanicolaou A, Yassour M, Grabherr M, Blood PD, Bowden J, Couger MB, Eccles D, Li B, Lieber M (2013). De novo transcript sequence reconstruction from RNA-seq using the Trinity platform for reference generation and analysis. Nat Protoc.

[30] Anders S, Huber W (2010). Differential expression analysis for sequence count data. Genome Biol.

[31] Bengert P, Dandekar T (2003). A software tool-box for analysis of regulatory RNA elements. Nucleic Acids Res.

[32] Dai X, Zhao PX (2011). psRNATarget: a plant small RNA target analysis server. Nucleic Acids Res.

[33] Feng H, Wang B, Zhang Q, Fu Y, Huang L, Wang X, Kang Z (2015). Exploration of microRNAs and their targets engaging in the resistance interaction between wheat and stripe rust. Front Plant Sci.

[34] Xin M, Wang Y, Yao Y, Xie C, Peng H, Ni Z, Sun Q (2010). Diverse set of microRNAs are responsive to powdery mildew infection and heat stress in wheat (Triticum aestivum L.). BMC Plant Biol.

[35] Wu HJ, Wang ZM, Wang M, Wang XJ (2013). Widespread long noncoding RNAs as endogenous target mimics for microRNAs in plants. Plant Physiol.

[36] De Lucia F, Dean C (2011). Long non-coding RNAs and chromatin regulation. Curr Opin Plant Biol.

[37] Pandey RR, Mondal T, Mohammad F, Enroth S, Redrup L, Komorowski J, Nagano T, Mancini-Dinardo D, Kanduri C (2008). Kcnq1ot1 antisense noncoding RNA mediates lineage-specific transcriptional silencing through chromatin-level regulation. Mol Cell.

[38] Kapranov P, Cheng J, Dike S, Nix DA, Duttagupta R, Willingham AT, Stadler PF, Hertel J, Hackermuller J, Hofacker IL (2007). RNA maps reveal new RNA classes and a possible function for pervasive transcription. Science.

[39] Llave C (2002). Endogenous and silencing-associated small RNAs in plants. Plant Cell Online.

[40] Maniatis T, Reed R (1987). The role of small nuclear ribonucleoprotein particles in pre-mRNA splicing. Nature.

[41] Black DL (2005). A simple answer for a splicing conundrum. Proc Natl Acad Sci U S A.

[42] Guo Z, Karunatilaka KS, Rueda D (2009). Single-molecule analysis of protein-free U2-U6 snRNAs. Nat Struct Mol Biol.

[43] Matera AG, Terns RM, Terns MP (2007). Non-coding RNAs: lessons from the small nuclear and small nucleolar RNAs. Nat Rev Mol Cell Biol.

[44] Urlaub H, Raker VA, Kostka S, Luhrmann R (2001). Sm protein–Sm site RNA interactions within the inner ring of the spliceosomal snRNP core structure. EMBO J.

[45] Wang P, Palfi Z, Preusser C, Lucke S, Lane WS, Kambach C, Bindereif A (2006). Sm core variation in spliceosomal small nuclear ribonucleoproteins from Trypanosoma brucei. EMBO J.

[46] Li Z, Huang C, Bao C, Chen L, Lin M, Wang X, Zhong G, Yu B, Hu W, Dai L (2015). Exon-intron circular RNAs regulate transcription in the nucleus. Nat Struct Mol Biol.

[47] Memczak S, Jens M, Elefsinioti A, Torti F, Krueger J, Rybak A, Maier L, Mackowiak SD, Gregersen LH, Munschauer M (2013). Circular RNAs are a large class of animal RNAs with regulatory potency. Nature.

[48] Huttenhofer A, Schattner P (2006). The principles of guiding by RNA: chimeric RNA-protein enzymes. Nat Rev Genet.

[49] Juan L, Wang G, Radovich M, Schneider BP, Clare SE, Wang Y, Liu Y (2013). Potential roles of microRNAs in regulating long intergenic noncoding RNAs. BMC Med Genet.

[50] Franco-Zorrilla JM, Valli A, Todesco M, Mateos I, Puga MI, Rubio-Somoza I, Leyva A, Weigel D, Garcia JA, Paz-Ares J (2007). Target mimicry provides a new mechanism for regulation of microRNA activity. Nat Genet.

