# Supplementary material for: Genome-wide identification and functional prediction of novel and fungi-responsive lincRNAs in Triticum aestivum
Source: BMC Genomics. 2016 Mar 15;17:238. doi: 10.1186/s12864-016-2570-0 (PMC4791882; doi:10.1186/s12864-016-2570-0)
Supplement: Additional file 1: Table S1. — SnRNP motif of wheat lincRNAs detected by RNA Analyzer. Table S2. The list of DE-LincRNAs as putative targets of miRNAs and its sequences. Table S3. Key miRNA target of functional gene at sense and antisense strand. Table S4. PCR primers used for Q-PCR amplification of DE-lincRNA. (PDF 857 kb) [file 12864_2016_2570_MOESM1_ESM.pdf]

Table S1. SnRNP motif of wheat lincRNAs detected by RNA Analyzer.

sequences	quality	sequences	quality	sequences	quality
aauuuuuguga	+	aguuuguag	+	aguuucuaa	+
aauuuguga	+	aauugugg	+	aguuuguuuugg	+
aguguugg	+	aauucugg	+	gauuucugg	+
gaucuuga	+	gaucuuag	+	aguuauuga	+
gguuguuga	+	aauuuguuaa	+	gguucuuga	+
aauuuuauga	+	gguucuag	+	gguuuguag	+
aguuuucuuaa	+	gauuuucuuuuaa	+	aauuucuag	+
aguguuuuuuag	+	gauucuuga	+	aguguuuuga	+
gguguugg	+	gguucuga	+	gauguuuuga	+
aguuuuguag	+	aguguuuga	+	aguuuuuuguuaa	+
gguguuaa	+	gguuuauag	+	aauguugg	+
aauguuag	+	aauuguugg	+	aguuauuugg	+
aauucuugg	+	aauuauugg	+	aguucuuag	+
gaucuugg	+	aauauuuuga	+	gguuauga	+
aguauuuaa	+	gauguuaa	+	aauuuuucuga	+
aaucuuga	+	gauucuga	+	*gauuuucuuuuuugg	+
gguauuuuga	+	aauguuuuga	+	aauuuauaa	+
aaucuuugg	+	aguuguuga	+	aauuuuuuucuga	+
gguguuugg	+	aaucuuuag	+	aauuuuauaa	+
aauuuuuaugg	+	aauuuauuaa	+	aguuguugg	+
gguuaugg	+	aguguuag	+	gauuaugg	+
aauucuuag	+	gguuucuaa	+	aauucuuuuga	+
aguucuugg	+	gguguuuaa	+	agucuuaa	+
gauuuuguuga	+	aauuuguuag	+	aauuuauuuga	+
aauuuguuuuaa	+	gguuuauaa	+	gauucuuaa	+
gauuuucuugg	+	aguuuuucuag	+	ggucuuuuaa	+
aguuuguaa	+	aauauuuag	+	aauuuauuuag	+
aaucuugg	+	ggucuuaa	+	gguuucuuga	+
gauguuag	+	aauuguuaa	+	aguuuuuucuaa	+
aauuucuga	+	gauuuguag	+	gauuucuugg	+
aauuucugg	+	gguguuuag	+	aaucuuuuag	+
aauuauuga	+	aguauugg	+	aguuuguuga	+
aauuuguag	+	gguuuguuuaa	+	gauuuauga	+
gauuguuugg	+	aauuuuuuuuguag	+	aauauuuuga	+
aauguuaa	+	aguuuuguuuugg	+	gauuguuuga	+
aauguuuga	+	gguuuguuga	+	aauuguuag	+
gguugugg	+	aguauuuag	+	gguuuucuuuuag	+
aguucuga	+	gaucuuuugg	+	aaucuuuaa	+
gauucuuuga	+	gauauuuuga	+	gguguuuuuuaa	+
gguuuauuuga	+	gguuuguaa	+	aguguuuaa	+
aguuuuauuugg	+	gguuuucuga	+	gauucugg	+
aguugugg	+	aguucuuaa	+	gauauuuuag	+
gauucuaa	+	aguuguga	+	gauuuauaa	+
aguucuag	+	gguucuuugg	+	gguuucuuag	+
aauauuuuaa	+	aauauuuuuuga	+	aauuauuuuaa	+
gguauuga	+	gguauuuugg	+	gguuuuuuauaa	+
aguuauaa	+	gguuuuauag	+	gguguuuuag	+
gauuucuuga	+	gauucuuag	+	gauauuuuuag	+

aauuucuaa	+	aguuuuauga	+	gguauugg	+
aauauuugg	+	aaucuuaa	+	aguauuaa	+
aauuguuuag	+	gauugugg	+	aguguuuuag	+
gguauuugg	+	gauauuugg	+	gauuauuaa	+
aguauuag	+	gauauugg	+	gguuuauugg	+
gauuguga	+	aauguuga	+	gguauuuag	+
gauucuag	+	gauauuuaa	+	aguuuucuuuaa	+
gguuugugg	+	aguuuauga	+	gguuuuguuuaa	+
gguuuuucugg	+	aauuguuuaa	+	gguguuuuaa	+
gauauuuag	+	gauguuag	+	gguuuguuag	+
gaucuuuag	+	aguauuga	+	aauguuuuuaa	+
ggucuuga	+	gauuauaa	+	aguuguuuugg	+
gauauuag	+	aauuuguaa	+	gguuguag	+
aguuauag	+	aguuuguga	+	gaucuuuuuag	+
gauuuuguag	+	gguuauag	+	ggucuuag	+
aguuuuguugg	+	aauucuag	+	aguucuuuaa	+
gguuuguga	+	gauuuuauaa	+	gguauuuga	+
aauuguag	+	gauuuuauuuaa	+	aauguuugg	+
gauauuaa	+	aguuguag	+	aauguuuuugg	+
aauauuaa	+	gguuuuuuauugg	+	aauauuuuag	+
aguuauga	+	gauuauuuga	+	aauauuuga	+
aguguuga	+	aguguuaa	+	agucuuuuuga	+
gaucuuuga	+	aauauuuuuag	+	gauuauuuuga	+
gauguuga	+	aguuuauuga	+	aauuuauugg	+
aauuuucuuga	+	aguucuaa	+	aguuauugg	+
aaucuuag	+	aauauugg	+	gauguuuga	+
gguguuga	+	gguuguga	+	gguuauuaa	+
aauucuaa	+	gguuauuag	+	gguuuucuuuga	+
gaucuuaa	+	aauucuuga	+	gauguuugg	+
aauauuga	+	aguuuuauag	+	gguucugg	+
aauuguga	+	gguuuuguga	+	gguguuag	+
agucuugg	+	aauauuag	+	gauguuuuag	+
aauuaugg	+	gauuguuuaa	+	ggucuuuga	+
aguucugg	+	gauauuga	+	gauuuucuuga	+
gauuguuuuag	+	gauuguuga	+	aauuuuuuauag	+
aauuauuugg	+	gguuguaa	+	gauuauga	+
gguuauaa	+	aauuuuuuuauuga	+	aauuuauuga	+
gguucuaa	+	aauuugugg	+	agucuuag	+
aauuauaa	+	gauuuuuguuga	+	aguucuuga	+
gaucuuuuuuuaa	+	aauuucuuag	+	gguuucuag	+
aguuucuag	+	ggucuuugg	+	aauuauuuga	+
gguauuag	+	aguuuucuga	+	gauuugugg	+
aauauuuaa	+	gguauuuuuuuag	+	gguucuugg	+
aauuuaugg	+	aguuuguuag	+	gguuuuuucuga	+
aauuuauag	+	gauuguugg	+	aauuuuauuaa	+
aauuauag	+	gauguugg	+	gauauuuga	+
aguuguaa	+	gauuauuuag	+	agucuuugg	+
aguuaugg	+	aauuuauga	+	aguuuauaa	+
gguauuaa	+	aguauuuuag	+	gauuauag	+
aauuauga	+	aauuuuauuga	+	aauuuuuuucuaa	+
aauuguaa	+	aauucuga	+		

aauuuuguaa	+	gauuuuuguuuag	+	gauuuucugg	+
gguuuguuuuuugg	+	aguucuuuga	+	aguuauuuuag	+
aguuuucuuag	+	aguuuaugg	+	gauuuuuauaa	+
gauuguag	+	aauguuuag	+	gguauuuuuuuga	+
aguuuuuucuga	+	aauuuuauugg	+	aauuuuguag	+
agucuuga	+	aaucuuuga	+	gauauuuuuga	+
aauuauuag	+	aguuuuguuuag	+	aguuguuuuag	+
aguuguuag	+	aguuuauag	+	aauuuucuaa	+
gguuuauuuaa	+	aguuauuag	+	gguguuuuuag	+
gauuauugg	+	aauuuuguga	+	*gauuuuuuguuuuaa	+
gguuguuuuag	+	aauuuugugg	+	aguuuuuuuguuaa	+
gauuguaa	+	aguuauuuuaa	+	aaucuuuuuaa	+
gguuuuauugg	+	gaucuuuaa	+	aguuguuugg	+
gauuuuuguag	+	gguuuuguugg	+	gauuauuuaa	+
aguauuuga	+	gguuguuuuuag	+	gauguuuugg	+
gguuuuuauga	+	gauuuguaa	+	gauguuuag	+
aauuauuaa	+	gguuuucuag	+	gguuuauuaa	+
gguuauuuga	+	gguuucugg	+	gauauuuuaa	+
agucuuuga	+	aauuguuga	+	agucuuuuuaa	+
aauuuguuuuag	+	aauucuuuag	+	gauuauuag	+
aauguuuugg	+	gauuucuga	+	aguuuguuaa	+
gguuguugg	+	gauucuuuag	+	aauguuuuuag	+
aguuucuuaa	+	aauuuguugg	+	aauuuucuuuaa	+
gauuuuucuag	+	aguuucuuugg	+	gauuucuaa	+
gauguuuaa	+	gguuauuuaa	+	gauuuuaugg	+
aauuuuguuaa	+	aguguuugg	+	gauuguuuag	+
gguuuuuauuaa	+	gauuuuguuugg	+	aaucuuuuga	+
aauuuuauag	+	aguuucuuga	+	aauuuuauuuuuga	+
aguuucugg	+	aauuuguuga	+	aauuucuuuuaa	+
gguuuuguaa	+	*gauuuuuuuuucuaa	+	gguuauuugg	+
aaucuuuuuugg	+	gauuuaugg	+	gauuuauag	+
gguuuuguag	+	aguuguuuga	+	aauuauuuugg	+
aauguuuuaa	+	aguuauuuuuuga	+	gauuuucuga	+
aguuucuga	+	gguuuauuugg	+	aauuuucuga	+
gauuuguga	+	aguuauuaa	+	gauuucuuag	+
aauauuuugg	+	aauuauuuaa	+	aauucuuuaa	+
aguuuauugg	+	aauuguuuga	+		

Table S2. DE-LincRNAs as putative targets of miRNAs (sense strand)

miRNA Acc.	Target Acc.	Expectation (E)	Target Accessibility (UPE)	Alignment	Inhibition	Multiplicity
aqc-miR395a	T13_Unigene_BMK.13743	3.0	6.74	miRNA 20 UCAAGGAGGUUUGGGAAGUC 1<br>: :::::: ::::::::::<br>Target 333 ACUUCCUCACAACCCUUCAG 352	Translation	1
ath-miR169a	T16_Unigene_BMK.63733	3.0	19.865	miRNA 21 AGCCGUUCAGUAGGAACCGAC 1<br>:::::: : .::::::::.::<br>Target 125 UCGGCAUGGUAUCCUUGGUUG 145	Cleavage	1
ath-miR2936	T16_Unigene_BMK.89858	3.0	18.294	miRNA 20 AGACACAAGAGAGAGAGUUC 1<br>::::::.::::::::.::<br>Target 290 AAUGUGUUUUCUCUCUCGAG 309	Cleavage	1
ath-miR319c	T13_Unigene_BMK.42814	3.0	20.696	miRNA 20 UCCUCGAGGGAAGUCAGGUU 1<br>:: ::: .::::::::.:::<br>Target 98 AGCAGCCUCCUUCAGUUCAA 117	Cleavage	1
ath-miR414	T13_Unigene_BMK.49993	1.5	6.866	miRNA 21 ACUGCUACUACUACUUCUACU 1<br>::.:::::::::: :::::::<br>Target 33 UGGCGAUGAUGAUAAAGAUGA 53	Cleavage	2
ath-miR414	T13_Unigene_BMK.49993	2.0	7.477	miRNA 21 ACUGCUACUACUACUUCUACU 1<br>:::.:::::::::::::: ::<br>Target 3 UGAUGAUGAUGAUGAAGAGGA 23	Cleavage	2
ath-miR414	T13_Unigene_BMK.33064	2.0	3.471	miRNA 21 ACUGCUACUACUACUUCUACU 1<br>:::.::::::::::: :::::<br>Target 4 UGAUGAUGAUGAUGAUGAUGA 24	Cleavage	1
ath-miR5012	T19_Unigene_BMK.51118	3.0	11.74	miRNA 20 CUUGUGUUCAUCGUCAUUUU 1<br>:::: ..:::.:.:::::::<br>Target 256 GAACUUGAGUGGUAGUAAAA 275	Cleavage	1

ath-miR5649a	T16_Unigene_BMK.8598	3.0	18.16	miRNA 21 UAUCAUUGGUUGUAUAAGUUA 1<br>.::::::.::: ::.:::::<br>Target 57 GUAGUAAUCAAAAUGUUCAAA 77	Translation	1
ath-miR773a	T10_Unigene_BMK.61842	3.0	15.47	miRNA 21 CUCUGUUUUCGACCUUCGUUU 1<br>::::.::::: : :.::::::<br>Target 306 GAGAUAAAAGGUAGGAGCAAA 326	Translation	1
ath-miR841b-3p	T10_Unigene_BMK.72915	3.0	13.734	miRNA 21 UUAUGCUGGGUGAUCUUUAAC 1<br>:.:..:::.::::.:::.:::<br>Target 974 AGUGUGACUCACUGGAAGUUG 994	Cleavage	1
bdi-miR1135	T4_Unigene_BMK.40145	3.0	17.485	miRNA 20 CAGCCUUAAUGAACAGCUUU 1<br>:::::: :::::::.:::.<br>Target 382 UUCGGAAAUACUUGUUGAAG 401	Cleavage	1
bdi-miR160d-3p	T1_Unigene_BMK.48244	3.0	21.065	miRNA 21 AUACGAACCGAGGCACGUGCG 1<br>:::: :::: :.::::::.::<br>Target 88 UAUGAUUGGGUUCGUGCAUGC 108	Cleavage	1
bdi-miR5066	T16_Unigene_BMK.67438	3.0	14.957	miRNA 21 UCCGUGAGGUGAAUAUGUGAA 1<br>: ::: ::::: :::::::::<br>Target 160 AAGCAAUCCACAUAUACACUU 180	Translation	1
bdi-miR5174b-5p	T19_Unigene_BMK.15126	2.0	11.716	miRNA 20 GUUAGAAAUACCUUGUCUCC 1<br>.: ::::::::::::::.::<br>Target 204 UAUUCUUUAUGGAACAGGGG 223	Cleavage	1
bdi-miR5174e-5p.2	T4_Unigene_BMK.2208	2.5	15.927	miRNA 20 AAAUACCUUGUCUCCCUCAU 1<br>:: ::::::.::::::::<br>Target 4 AUUUUGGAACGGAGGGAGUA 23	Cleavage	1
bdi-miR7723b-5p	T13_Unigene_BMK.17881	3.0	12.681	miRNA 24 ACUACUUCCAUCAUCAUAGUUUUA 1<br>: : :.::::.::.: ::.:::::<br>Target 490 UAAAGGAGGUGGUGGAAUUAAAAU 513	Translation	1

bdi-miR7726a-5p	T19_Unigene_BMK.2493	3.0	16.864	miRNA 20 GCUGCAGGCAUGGUAGUAGU 1<br>:: :::.::: .::::::::<br>Target 90 CGUCGUUCGUUUCAUCAUCA 109	Translation	1
bdi-miR7765-3p	T13_Unigene_BMK.17881	3.0	16.611	miRNA 20 AAAUCAGGUUGAAGAACAUU 1<br>:::::. :::::.::::::<br>Target 1342 CUUAGUUAAACUUUUUGUAA 1361	Cleavage	1
bra-miR5725	T13_Unigene_BMK.83254	3.0	15.046	miRNA 21 CGUCUAGU-CUAACACGGUUUA 1<br>:::::::: :::::::.::.::<br>Target 750 GCAGAUCAUGAUUGUGUCAGAU 771	Cleavage	1
cca-miR6115	T16_Unigene_BMK.32266	3.0	20.729	miRNA 21 GUGUACACGUAUGGCAGGUCU 1<br>.::: :::::: .::::.:::<br>Target 1082 UACAAGUGCAUCUCGUCUAGA 1102	Translation	1
cre-miR1165-3p	T19_Unigene_BMK.31149	3.0	24.523	miRNA 20 AUAGGCAUGUUCGCCAGGCA 1<br>:: :.: ::.::::::::::<br>Target 275 UAGCUGCACGAGCGGUCCGU 294	Cleavage	1
esi-miR3454c-3p	T16_Unigene_BMK.13852	2.0	13.718	miRNA 20 GAUGGGUCGAGGCUACCUUC 1<br>::.:::::::. ::::::::<br>Target 768 CUGCCCAGCUUGGAUGGAAG 787	Translation	1
ghr-miR7502	T10_Unigene_BMK.70689	3.0	13.886	miRNA 21 UAAGUAAAGAUGACAAUUUUU 1<br>.::.::::::::::::: .:.<br>Target 90 GUUUAUUUCUACUGUUAUGAG 110	Cleavage	1
gma-miR1535b	T19_Unigene_BMK.12077	3.0	16.584	miRNA 20 AUCUGUAGUGGUGUUUGUUC 1<br>: :.:::::::::.:..:::<br>Target 406 UUGGCAUCACCACGAGUAAG 425	Cleavage	1
gma-miR408a-5p	T7_Unigene_BMK.43633	3.0	20.405	miRNA 20 UACGAGACGGACAAGGGGAC 1<br>.: :::::.: :::::::::<br>Target 297 GUUCUCUGUCGGUUCCCCUG 316	Translation	1

gma-miR4396	T13_Unigene_BMK.18884	3.0	10.051	miRNA 22 GUCGUAGCAGAAUCUUUGAUGU 1<br>..: ::.::.::::::.::.::<br>Target 6 UGGGAUUGUUUUAGAAGCUGCA 27	Cleavage	1
gma-miR4407	T19_Unigene_BMK.55749	3.0	20.213	miRNA 20 AUGUUCACGACGAAGGAGAC 1<br>::: :::::::::::.:.:<br>Target 544 UACUAGUGCUGCUUCUUUUU 563	Cleavage	1
gma-miR482b-3p	T13_Unigene_BMK.17661	3.0	18.567	miRNA 20 AUACCCUCCACAUCCCUUCU 1<br>: :::: :::::.::::::.<br>Target 369 UUUGGGUGGUGUGGGGAAGG 388	Cleavage	1
gma-miR4992	T13_Unigene_BMK.29048	2.5	8.119	miRNA 21 AUUGUUUUUGGUAGAAUCUUA 1<br>::::::::.:::::::: .::<br>Target 13 UAACAAAAGCCAUCUUAAGAU 33	Cleavage	1
gma-miR5037a	T10_Unigene_BMK.9590	3.0	16.664	miRNA 20 CAUCACCUUCGGAAACUCCG 1<br>:::: ::::..::::::::<br>Target 480 GUAGAUGAAGUUUUUGAGGC 499	Cleavage	1
gma-miR5781	T16_Unigene_BMK.68333	0.5	15.659	miRNA 21 GUCUACGUCAGAGUCAAAGUC 1<br>.:::::::::.::::::::::<br>Target 63 UAGAUGCAGUUUCAGUUUCAG 83	Cleavage	1
hbr-miR6169	T10_Unigene_BMK.68603	3.0	15.726	miRNA 21 UCUCUCUUUUCUCUUUAUGAU 1<br>:::::::.:: ::::: ::::<br>Target 213 AGAGAGAGAACAGAAAAACUA 233	Translation	1
hme-miR-279c	T13_Unigene_BMK.25967	3.0	16.919	miRNA 22 UCCUAUUCACUUUUAGAUCAGU 1<br>::: :::: ::::: :::::::<br>Target 314 AGGUUAAGAGAAAAACUAGUCA 335	Cleavage	1
hme-miR-305	T10_Unigene_BMK.7014	3.0	15.071	miRNA 20 UCGUGGACUACUUCAUGUUA 1<br>::::: ::::::::..:::<br>Target 683 AGCACAAGAUGAAGUGUAAU 702	Cleavage	1

hme-miR-9b	T16_Unigene_BMK.6110	3.0	5.136	miRNA 22 GUAUGUCGAUCUUUUGG-UUUCU 1<br>:. :.:::::::::::: :::::<br>Target 58 CGCAUAGCUAGAAAACCUAAAGA 80	Cleavage	1
hvu-miR5049e	T10_Unigene_BMK.64534	3.0	6.445	miRNA 20 GGGAGACAUGGAUUUAUUAA 1<br>.::: : ::::::::::.::<br>Target 267 UCCUAUAUACCUAAAUAGUU 286	Cleavage	1
hvu-miR6195	T16_Unigene_BMK.13852	3.0	12.158	miRNA 21 GAGUAGGGAUGAUGCAUGAGU 1<br>.:: :::::::::.:::: ::<br>Target 847 UUCUUCCCUACUAUGUACACA 867	Cleavage	1
mdm-miR164a	T13_Unigene_BMK.82017	3.0	18.903	miRNA 20 CGUACACGGGACGAAGAGGU 1<br>:: :::::.::::::::.:<br>Target 215 GCCUGUGCUCUGCUUCUUCU 234	Cleavage	1
mdm-miR169e	T4_Unigene_BMK.31722	3.0	7.257	miRNA 22 GGUUUGUUGCGAGAAGAGAAGU 1<br>...::.::. :::::::.::::<br>Target 613 UUGAAUAAUUCUCUUCUUUUCA 634	Cleavage	1
mtr-miR2634	T13_Unigene_BMK.34604	2.5	14.803	miRNA 20 UCGUUGUUUGACUCUUAUUU 1<br>:: :: :.::::::::::::<br>Target 1705 AGAAAAAGACUGAGAAUAAA 1724	Cleavage	1
mtr-miR2649	T16_Unigene_BMK.5516	3.0	12.049	miRNA 20 GUGAGUAUUAACCGUGGAAA 1<br>:.: .:::::: ::::::::<br>Target 387 CGCCUAUAAUUAGCACCUUU 406	Translation	1
mtr-miR5237	T16_Unigene_BMK.78117	2.5	12.641	miRNA 20 AGGGUUGAUUUAGAAAACUU 1<br>:.:.:::::.:: :::::::<br>Target 790 UUCUAACUAGAUAUUUUGAA 809	Cleavage	1
mtr-miR5558-5p	T4_Unigene_BMK.36197	3.0	10.142	miRNA 20 UAUCUGAAUCUUAACCUUUU 1<br>: ::::::: ::::::::::<br>Target 117 AGAGACUUA-AAUUGGAAAA 135	Translation	1

mtr-miR5760	T10_Unigene_BMK.67416	2.5	12.849	miRNA 24 AGGAAUUGUUUAUAGGAAUUUCGU 1<br>::.:::::::: :: ::::::::.<br>Target 419 UCUUUAACAAAAAUGCUUAAAGCG 442	Translation	1
nta-miR6150	T16_Unigene_BMK.17652	3.0	17.498	miRNA 22 CGGUUCUGCUAGUUUGUUUAGA 1<br>:. ::::::::::...:.::::<br>Target 904 GUGAAGACGAUCAGGUAGAUCU 925	Cleavage	1
nta-miR6155	T10_Unigene_BMK.69095	3.0	17.208	miRNA 20 CGUUCUCGUUCCGUUGGAAU 1<br>::.::::: ::.:::::::<br>Target 80 CCAGGAGCAUGGUAACCUUA 99	Translation	1
osa-miR1319a	T19_Unigene_BMK.44304	3.0	13.617	miRNA 20 UAUAUAAUGUCUACGGCCAA 1<br>::.:: ::::: :::.::::<br>Target 465 AUGUACUACAGCUGCUGGUU 484	Translation	1
osa-miR1439	T19_Unigene_BMK.34869	2.0	11.606	miRNA 20 UAUGAGUGAGGCAAGGUUUU 1<br>:::::: :::.:::.:::::<br>Target 724 AUACUCCCUCUGUUUCAAAA 743	Cleavage	1
osa-miR1439	T16_Unigene_BMK.64464	3.0	8.425	miRNA 20 UAUGAGUGAGGCAAGGUUUU 1<br>.::::: ::::::::: :::<br>Target 1001 GUACUCCCUCCGUUCCCAAA 1020	Cleavage	1
osa-miR1873	T16_Unigene_BMK.67438	3.0	16.597	miRNA 24 GAAGGUCGAGACUAUGGUACAACU 1<br>: :.:.:::: ::::::::::: :<br>Target 94 CCUUCGGCUCGGAUACCAUGUUAA 117	Cleavage	1
osa-miR414	T13_Unigene_BMK.49993	1.5	7.477	miRNA 20 CUGCUACUACUACUCCUACU 1<br>::.:::::::: ::::::::<br>Target 7 GAUGAUGAUGAAGAGGAUGA 26	Translation	2
osa-miR5148a	T1_Unigene_BMK.23686	3.0	12.716	miRNA 24 UACUAUACUGUAAAGAUGGGGAGU 1<br>:: ::::::::: .::.:::::<br>Target 170 AUCUUAUGACAUUAUUAUCCCUCU 193	Translation	1

osa-miR5834	T13_Unigene_BMK.34604	3.0	10.323	miRNA 20 AGUGGUUAAAGAUGUAGGCA 1<br>:::.::::::: :::::.::<br>Target 1442 UCAUCAAUUUC-ACAUCUGU 1460	Translation	1
osa-miR815a	T19_Unigene_BMK.63822	3.0	19.791	miRNA 20 GGUUAGAGGAGUUAGGGGAA 1<br>::: .::.:::::::::::<br>Target 362 CCAUCUUCUUCAAUCCCCUU 381	Cleavage	1
ppt-miR414	T13_Unigene_BMK.29133	3.0	16.703	miRNA 20 CUGCUCCUACUACUCCUACU 1<br>::.:::::::::: :::::<br>Target 677 GAUGAGGAUGAUGUGGAUGC 696	Cleavage	1
ptc-miR171g-5p	T10_Unigene_BMK.38424	3.0	21.718	miRNA 20 UACUAACUCGGUAGGGUUGU 1<br>:::::: .::::::::.:..<br>Target 193 AUGAUUCGGCCAUCCCGAUG 212	Cleavage	1
ptc-miR478d	T16_Unigene_BMK.16253	3.0	16.732	miRNA 20 AUUUUUAUCUUCUGUACAGU 1<br>:.:::: ::.:::..:::::<br>Target 480 UGAAAACAGGAGAUGUGUCA 499	Cleavage	1
ptc-miR482c-5p	T19_Unigene_BMK.25423	3.0	1.362	miRNA 20 AGUAAGGGCGGAGAGGGUAU 1<br>.:::::: .::::::::::<br>Target 72 CUAUUCCCAUCUCUCCCAUA 91	Cleavage	1
sbi-miR1435a	T16_Unigene_BMK.34607	3.0	20.512	miRNA 20 CUUUUCAAACUGAAUUCUUU 1<br>::::.: :::::::::.::<br>Target 105 GAAAGGGGUGACUUAAGGAA 124	Cleavage	1
sbi-miR5568f-3p	T16_Unigene_BMK.4332	3.0	20.687	miRNA 20 AGGUAAGGUUUAAUAUUCUG 1<br>:.::::.:: :::.::::.:<br>Target 259 UUCAUUUCACAUUGUAAGGC 278	Translation	1
stu-miR398a-5p	T19_Unigene_BMK.42350	3.0	22.377	miRNA 20 UAUACAAGAGUUUAGUUGGG 1<br>: ::::::::: ::::.:.:<br>Target 65 AAAUGUUCUCACAUCAGCUC 84	Translation	1

stu-miR7122-5p	T13_Unigene_BMK.14637	2.5	14.344	miRNA 20 UGUCGCCAAAGAGACAUAUU 1<br>: :::::: :::.:::::::<br>Target 772 AAAGCGGUCUCUUUGUAUAA 791	Cleavage	1
tae-miR1133	T19_Unigene_BMK.3424	3.0	17.644	miRNA 22 AAAGCCU-GCCUCCCUCAUAUAC 1<br>::: ::: :::::::::::::<br>Target 2 UUUGGGAACGGAGGGAGUAUAUG 24	Cleavage	1
zma-miR167j-3p	T1_Unigene_BMK.23686	3.0	14.038	miRNA 20 UUACUUUGACGGUGUACUAG 1<br>:.:::::::::: ::: :::<br>Target 594 AGUGAAACUGCCUCAUCAUC 613	Cleavage	1
zma-miR2275d-3p	T16_Unigene_BMK.22835	3.0	6.062	miRNA 20 UCUAUAAUCUCCUUUUGUUU 1<br>:::::::::: :::::..:<br>Target 84 AGAUAUUAGAUUAAAACGGA 103	Translation	1

Table S2. DE-LincRNAs as putative targets of miRNAs (antisense strand)

miRNA Acc.	Target Acc.	Expectation (E)	Target Accessibility (UPE)	Alignment	Inhibition	Multiplicity
aly-miR169d-5p	T16_Unigene_BMK.16253	3.0	16.991	miRNA 20 CCGUUCAGUAGGAACCGAGU 1<br>: :: ::::::::::::::<br>Target 149 AGAAAUUCAUCCUUGGCUCA 168	Cleavage	1
aly-miR3449-3p	T16_Unigene_BMK.16253	2.5	16.191	miRNA 20 UAGGUCUAACGAAAGAUAGA 1<br>::::: ::::::::: ::::<br>Target 84 AUCCAUAUUGCUUUCCAUCU 103	Cleavage	1
aly-miR3446-3p	T10_Unigene_BMK.48402	3.0	19.101	miRNA 22 AAGCCUUCGAUUUUCUCACCGU 1<br>: .:::: ::.::: :::::::<br>Target 58 UCUGGAAACUGAAACAGUGGCA 79	Cleavage	1
aly-miR835-5p	T19_Unigene_BMK.46503	3.0	9.413	miRNA 21 CUAUUUCUUGUAUACGUUCUU 1<br>::::: .:::::::.:::::<br>Target 328 GAUAACUGACAUAUGUAAGAA 348	Cleavage	1

aly-miR837-5p	T13_Unigene_BMK.15115	3.0	15.254	miRNA 20 CUUUUUUUGUUCUUUGUUAC 1<br>::.:::. :::::::::::<br>Target 495 AAAGAAAGAAAGAAACAAUG 514	Cleavage	1
ath-miR426	T13_Unigene_BMK.33891	3.0	6.05	miRNA 20 CAUUCCUGUUUAAAGGUUUU 1<br>:::: ::::.::::.::::<br>Target 1056 CUAAGUACAAGUUUCUAAAA 1075	Cleavage	1
ath-miR5023	T13_Unigene_BMK.22353	3.0	16.333	miRNA 20 GGGGGAAUAGGUGAUGGUUA 1<br>:. :.::::::::::..:::<br>Target 189 CUACUUUAUCCACUAUUAAU 208	Cleavage	1
ath-miR5025	T19_Unigene_BMK.44037	3.0	12.017	miRNA 20 CAGUGAAUGUAUAUAUGUCA 1<br>::::: :..: :::::::::<br>Target 17 GUCACCUGUAAAUAUACAGU 36	Translation	1
ath-miR5631	T13_Unigene_BMK.39161	3.0	15.508	miRNA 20 UUUAAUACAGAAAGGACGGU 1<br>::::.:::. :::::::::<br>Target 201 UAAUUGUGUUGUUCCUGCCA 220	Translation	1
ath-miR5635a	T4_Unigene_BMK.31722	3.0	8.297	miRNA 20 UGGCAAUUGUGAGGAAUUGU 1<br>.:::::: .:.:.:::::::<br>Target 583 GCCGUUAUUAUUUCUUAACA 602	Cleavage	1
ath-miR5658	T13_Unigene_BMK.33064	1.0	0.181	miRNA 20 AAGUAGUAGUAGUAGUAGUA 1<br>:::::::::::::::::::<br>Target 451 AUCAUCAUCAUCAUCAUCAU 470	Cleavage	1
ath-miR5658	T13_Unigene_BMK.49993	3.0	3.944	miRNA 21 AAAGUAGUAGUAGUAGUAGUA 1<br>::::.:::::::: ::::: :<br>Target 331 UUUCGUCAUCAUCCUCAUCUU 351	Cleavage	1
ath-miR5658	T10_Unigene_BMK.7014	3.0	10.334	miRNA 21 AAAGUAGUAGUAGUAGUAGUA 1<br>: :.::.:::::.:::::.::<br>Target 548 UCUUAUUAUCAUUAUCAUUAU 568	Cleavage	1

ath-miR5665	T13_Unigene_BMK.26060	3.0	10.698	miRNA 21 UAGGGUCUAGAACAGGUGGUU 1<br>::::.::::.:: ::..::::<br>Target 1239 AUCCUAGAUUUUAUCUGCCAA 1259	Translation	1
ath-miR829.1	T16_Unigene_BMK.29292	3.0	9.256	miRNA 24 UAAGGUAGUAAACCAUAGUCUCGA 1<br>: :.: ::: :::::::::::::<br>Target 2 ACUUCCUCACAUGGUAUCAGAGCU 25	Cleavage	1
bdi-miR1127	T16_Unigene_BMK.92879	3.0	17.81	miRNA 20 UAGCCUGCCUCCCUCAUCAA 1<br>: ::::.:::::::::: ::<br>Target 29 ACCGGAUGGAGGGAGUAUUU 48	Cleavage	1
bdi-miR5065	T16_Unigene_BMK.67438	2.0	18.226	miRNA 21 UCACAUAUUCACUUAACGGAU 1<br>:::::::: :::.:::::.::<br>Target 1087 AGUGUAUAUGUGGAUUGCUUA 1107	Cleavage	1
bdi-miR5173-3p	T10_Unigene_BMK.78030	3.0	24.193	miRNA 20 AAGAGCAUAUAUGUCUACGU 1<br>::: :::::.::..::::.:<br>Target 241 UUCACGUAUGUAUGGAUGUA 260	Cleavage	1
bdi-miR5174e-3p.2	T4_Unigene_BMK.2208	2.5	15.199	miRNA 21 GAUGAGGGAGGCAAGGUAUUU 1<br>:::::::::::::::::: ::<br>Target 336 CUACUCCCUCCGUUCCAAAAU 356	Cleavage	1
bdi-miR5174e-5p.2	T19_Unigene_BMK.34869	3.0	11.763	miRNA 21 GAAAUACCUUGUCUCCCUCAU 1<br>: :: :: :::::::::::::<br>Target 56 CAUUUUGAAACAGAGGGAGUA 76	Cleavage	1
bdi-miR5185m-3p	T13_Unigene_BMK.13743	3.0	11.12	miRNA 20 GAAGAUCAAGUUAAAAGUUU 1<br>:::.:::::.: :::::::<br>Target 44 CUUUUAGUUUAUUUUUCAAC 63	Translation	1
bdi-miR7714-5p	T10_Unigene_BMK.64534	3.0	14.98	miRNA 22 CAUUAUAACUAGGCUCUUUUAU 1<br>::::: : :::..::::::::::<br>Target 830 GUAAUUUAGAUUUGAGAAAAUA 851	Cleavage	1

cme-miR854	T13_Unigene_BMK.33064	3.0	0.197	miRNA 21 GAGGAGGAGUGAUAGGAGUAG 1<br>::::::.:::: ::: :::::<br>Target 439 CUCCUCUUCACCAUCAUCAUC 459	Translation	1
cre-miR1173	T7_Unigene_BMK.40941	3.0	17.59	miRNA 21 GUACUAAAGAUAACGUUGGUA 1<br>.:::::::: :::::::::<br>Target 595 UAUGAUUUCGCGUGCAACCAU 615	Translation	1
esi-miR3453-5p	T16_Unigene_BMK.14974	3.0	24.202	miRNA 20 UCCGCCCGCUAGCUCCUCUU 1<br>.::.::: :::.:::::::.<br>Target 140 GGGUGGGAGAUUGAGGAGAG 159	Cleavage	1
esi-miR3454c-3p	T13_Unigene_BMK.26045	2.5	16.969	miRNA 20 GAUGGGUCGAGGCUACCUUC 1<br>:::.::.:.:..::::::::<br>Target 20 CUAUCCGGUUUUGAUGGAAG 39	Cleavage	1
ghr-miR2948-5p	T16_Unigene_BMK.10482	3.0	11.071	miRNA 21 AAGAACGGGUUGAGAGGGUGU 1<br>::::::::.: .:.:::.:::<br>Target 249 UUCUUGCCUAUUUUUCCUACA 269	Translation	1
gma-miR1520a	T7_Unigene_BMK.43633	3.0	14.399	miRNA 22 CUGACAGUACAUAGUACAAGAU 1<br>: ::::: :::.:.::::::::<br>Target 264 GUCUGUC-UGUGUUAUGUUCUA 284	Cleavage	1
gma-miR156g	T10_Unigene_BMK.68603	3.0	18.63	miRNA 20 GACACGAGAGAUAGAAGACA 1<br>:::: :::::: :.::.:::<br>Target 185 CUGUUCUCUCUCUUUUUUGU 204	Translation	1
gma-miR167h	T13_Unigene_BMK.33010	3.0	19.846	miRNA 20 CAACUUCGACGGUCGUACUA 1<br>:::::: ::.:::::::.:<br>Target 488 AUUGAAGAUGUCAGCAUGGU 507	Cleavage	1
gma-miR4380a	T16_Unigene_BMK.21355	3.0	15.989	miRNA 24 UACGUGUAUGCCUAGUUGUUAGGC 1<br>.: :::::: ::::: :.:.::::<br>Target 58 GUACACAUAAGGAUCCAUAGUCCG 81	Translation	1

gma-miR5378	T16_Unigene_BMK.83333	3.0	18.0	miRNA 20 ACACAAGAUAGGAAGUCUAC 1<br>::::::::.: . :::::::<br>Target 575 UGUGUUCUGUGUCUCAGAUG 594	Translation	1
gma-miR5379	T10_Unigene_BMK.81367	3.0	24.327	miRNA 22 AUAGUAUUACUUACUAAAAGUA 1<br>: :: : :::::::.::::.::<br>Target 697 UCUCUUCAUGAAUGGUUUUUAU 718	Cleavage	1
hme-miR-6309-3p	T13_Unigene_BMK.24210	2.5	8.572	miRNA 20 UUCAUCUUUUACUUCUGGUA 1<br>::: ::: ::::::::::.::<br>Target 561 AAGCAGACAAUGAAGACUAU 580	Cleavage	1
hvu-miR5049f	T1_Unigene_BMK.48244	1.0	10.555	miRNA 21 AGGGAGGCUAGGUAUAAUUAA 1<br>::::::: :::::::::::::<br>Target 1 UCCCUCCUAUCCAUAUUAAUU 21	Cleavage	1
hvu-miR6192	T13_Unigene_BMK.33010	3.0	15.573	miRNA 22 UCUAGGGAAGGGGGGAGAGGAU 1<br>: ::::: :::: :::::::::<br>Target 324 AUAUCCCCUCCCGCCUCUCCUU 345	Translation	1
hvu-miR6197	T19_Unigene_BMK.66363	3.0	14.725	miRNA 20 CAGAAUGUAAAUCCUUGUCU 1<br>: ::::.:::::: ::::::<br>Target 277 GGCUUAUAUUUAGAAACAGA 296	Cleavage	1
lja-miR7533a	T19_Unigene_BMK.49300	2.5	16.03	miRNA 20 GUCGAAGAGAGGUAGGGGAG 1<br>:: :::::::.::::.:::.<br>Target 164 CAUCUUCUCUUCAUCUCCUU 183	Cleavage	1
mtr-miR2199	T16_Unigene_BMK.4332	3.0	14.523	miRNA 20 ACUAGGCACGAUCACAUAGU 1<br>::::.: ::::.::::.:.:<br>Target 195 UGAUUCAUGCUGGUGUGUUA 214	Cleavage	1
mtr-miR2634	T4_Unigene_BMK.41720	3.0	21.851	miRNA 21 CUCGUUGUUUGACUCUUAUUU 1<br>:::::: :::::::.::.:::..<br>Target 93 GAGCAAGAAACUGGGAGUAGG 113	Cleavage	1

mtr-miR2644	T16_Unigene_BMK.94232	3.0	20.377	miRNA 21 UGUGUGGUAGUUAGACUUCAC 1<br>:.: :::::.:.:::::.:::<br>Target 261 AUAGACCAUUAGUCUGAGGUG 281	Cleavage	1
mtr-miR2662	T13_Unigene_BMK.83915	2.5	17.565	miRNA 21 UAAGCCAAGUGUAAAAAUGAG 1<br>:: .::::.:::::::::::.<br>Target 57 AUAUGGUUUACAUUUUUACUU 77	Cleavage	1
mtr-miR5235a	T10_Unigene_BMK.63275	3.0	7.129	miRNA 21 GUGCGGUUAGUAACUGGAAUA 1<br>:.: :.::::: :::::::::<br>Target 365 CGCACUAAUCACUGACCUUAU 385	Translation	1
mtr-miR5273	T4_Unigene_BMK.10971	3.0	11.881	miRNA 24 GGAGAAGAGUUUGAUGUCGGGGAU 1<br>:: .::.:.:..::::::..::::<br>Target 28 CCAUUUUUUAGGCUACAGUUCCUA 51	Cleavage	1
mtr-miR5290	T13_Unigene_BMK.49993	3.0	3.88	miRNA 20 ACAGAUAGAGAGAGGUUUAA 1<br>: ::::: ::: ::::::::<br>Target 313 UCUCUAUAUCUGUCCAAAUU 332	Translation	1
nta-miR6162	T13_Unigene_BMK.19448	3.0	18.245	miRNA 20 GGUUACCGAUGGUAAAAAGA 1<br>:::::::.: ...:::::::<br>Target 322 CCAAUGGUUCUUGUUUUUCU 341	Translation	1
osa-miR1437b-3p	T19_Unigene_BMK.47431	3.0	22.438	miRNA 22 GCCGUGGCCUCGAGCGGUCGUG 1<br>: ::::: :::::::::::::<br>Target 93 CCGCACCC-AGCUCGCCAGCAC 113	Cleavage	1
osa-miR1439	T4_Unigene_BMK.2208	2.0	15.199	miRNA 20 UAUGAGUGAGGCAAGGUUUU 1<br>::::: :::::::::::::<br>Target 336 CUACUCCCUCCGUUCCAAAA 355	Cleavage	1
osa-miR1439	T19_Unigene_BMK.3424	2.5	7.919	miRNA 20 UAUGAGUGAGGCAAGGUUUU 1<br>:::::: ::::::::: :::<br>Target 782 AUACUCCCUCCGUUCCCAAA 801	Cleavage	1

osa-miR1863a	T16_Unigene_BMK.29292	2.0	9.256	miRNA 20 AGAUUGUACCAUAGUCUCGA 1<br>:: ::::::::::::::::<br>Target 6 CCUCACAUGGUAUCAGAGCU 25	Cleavage	1
osa-miR2275d	T10_Unigene_BMK.74750	3.0	9.222	miRNA 20 UCUAUAACCUCUUUUUGUUC 1<br>.:: ::: :::::::.::::<br>Target 729 GGAAAUUAGAGAAAAGCAAG 748	Cleavage	1
osa-miR2864.1	T19_Unigene_BMK.25423	2.5	14.461	miRNA 20 CGUUUUGUUCCCGUCGUUUU 1<br>:::::::: ::.:::::::<br>Target 46 CCAAAACAAUGGUAGCAAAA 65	Translation	1
osa-miR2875	T19_Unigene_BMK.36312	2.0	12.315	miRNA 22 AUUUGACAUAUACUGACAUUUA 1<br>: :.::: ::.:::::::::::<br>Target 564 UCAGCUGCAUGUGACUGUAAAU 585	Cleavage	1
osa-miR2931	T16_Unigene_BMK.29097	3.0	7.899	miRNA 20 AAAACUGUAGUUGUUAUUUC 1<br>::: :::: ::::::::::<br>Target 826 UUUGAACAUAAACAAUAAAG 845	Translation	1
osa-miR444b.1	T10_Unigene_BMK.78590	3.0	14.252	miRNA 20 CGUCGUUCGAACUCUGUUGU 1<br>::::::.:::: ::::::.<br>Target 113 GCAGCAGGCUUAAGACAAUU 132	Translation	1
osa-miR5150-5p	T19_Unigene_BMK.42425	3.0	14.354	miRNA 23 UCUUUGACGUCGACAGUCUUCGA 1<br>: ::::: :::: :::::::::<br>Target 283 ACAAACUUCAGCAGUCAGAAGCA 305	Translation	1
osa-miR5539a	T16_Unigene_BMK.29292	2.0	6.945	miRNA 20 CGUGCGCGUAGGCAAAAGAA 1<br>::::::: :::::::::::<br>Target 500 UCACGCGCCUCCGUUUUCUU 519	Cleavage	1
osa-miR5809	T13_Unigene_BMK.86345	3.0	21.913	miRNA 20 CGACACCAGCGGCCGCUGCU 1<br>:::: :: .::::::::.::<br>Target 306 GCUGCGGAUGCCGGCGAUGA 325	Cleavage	1

pab-miR1863	T16_Unigene_BMK.29292	2.0	9.256	miRNA 20 AGAUUGUACCAUAGUCUCGA 1<br>:: ::::::::::::::::<br>Target 6 CCUCACAUGGUAUCAGAGCU 25	Cleavage	1
pde-miR947	T13_Unigene_BMK.40810	2.5	18.148	miRNA 21 UUUGUCAUUGUCUAAGGCUAC 1<br>::.:::::::::.::: ::::<br>Target 648 AAGCAGUAACAGGUUCAGAUG 668	Cleavage	1
ptc-miR1445	T16_Unigene_BMK.13521	3.0	15.84	miRNA 20 AAAAAGAUCAGAUGUUCCCU 1<br>:::::: :: ::::::::::<br>Target 498 UUUUUCCAG-CUACAAGGGA 516	Translation	1
ptc-miR169n-5p	T16_Unigene_BMK.16253	3.0	16.991	miRNA 20 CCGUUCAGUAGGAACCGAGU 1<br>: :: ::::::::::::::<br>Target 149 AGAAAUUCAUCCUUGGCUCA 168	Cleavage	1
ptc-miR6441	T13_Unigene_BMK.39161	3.0	10.72	miRNA 20 ACAUCAGGAAGGCAGUUGAA 1<br>::::::.: ::.:::.:.::<br>Target 374 UGUAGUUCCUCUGUCGAUUU 393	Cleavage	1
ptc-miR6441	T16_Unigene_BMK.78117	3.0	13.028	miRNA 20 ACAUCAGGAAGGCAGUUGAA 1<br>:::: ::::::: :.::.::<br>Target 87 UGUAUUCCUUCCUUUAAUUU 106	Cleavage	1
ptc-miR6468-5p	T10_Unigene_BMK.9590	3.0	16.436	miRNA 20 CCCUCACUAAGUCCCUUUUG 1<br>: :.:::.:: :::::::::<br>Target 334 GUGGGUGGUUAAGGGAAAAC 353	Translation	1
ptc-miR7817a	T19_Unigene_BMK.61316	2.0	21.294	miRNA 21 ACAGAGCUCUGUUAUUGGUUU 1<br>:::::::::.::. :::::::<br>Target 134 UGUCUCGAGGCAGCAACCAAA 154	Cleavage	1
pti-miR5472	T16_Unigene_BMK.26398	3.0	16.04	miRNA 21 GAACCUGAUCGCGUUGUAAAA 1<br>:: : :::::.:.::::::::<br>Target 547 CUAGCACUAGUGUAACAUUUU 567	Cleavage	1

smo-miR1109	T16_Unigene_BMK.10482	2.5	5.378	miRNA 20 AAUGUGUUUUAGAGGGUGAU 1<br>::::::::::: :::::.:<br>Target 198 UUACACAAAAUAUCCCAUUU 217	Translation	1
stu-miR1886h	T13_Unigene_BMK.29048	2.0	18.014	miRNA 20 UCUACUUUAGUUGCAUUUUA 1<br>:::::::.:...::::::::<br>Target 794 AGAUGAAGUUGGCGUAAAAU 813	Cleavage	1
stu-miR7982a	T16_Unigene_BMK.16515	3.0	9.305	miRNA 21 AUAUAAUAAUAGUAGGUUGAA 1<br>:::::: :::::::: ::.::<br>Target 54 UAUAUUUUUAUCAUCAAAUUU 74	Cleavage	1
stu-miR7994a	T19_Unigene_BMK.25513	3.0	18.63	miRNA 20 AAAUACGGGUUCAUAUUAUA 1<br>:::::::::: :::::::<br>Target 49 UUUAUGCCCACAAAUAAUAU 68	Translation	1
stu-miR8045	T13_Unigene_BMK.29133	3.0	10.184	miRNA 20 UUGUGU-GGAGUUGAUAGUUA 1<br>:.:::: ::::::.:::::::<br>Target 473 AGCACAGCCUCAAUUAUCAAU 493	Cleavage	1
tae-miR1137	T16_Unigene_BMK.13521	0.0	14.737	miRNA 20 CUACUGAGUUGAAACAUGAU 1<br>::::::::::::::::::::<br>Target 2 GAUGACUCAACUUUGUACUA 21	Cleavage	1
tae-miR1137	T16_Unigene_BMK.63733	2.0	3.478	miRNA 20 CUACUGAGUUGAAACAUGAU 1<br>:: ::::::::::::::::<br>Target 648 AAUUACUCAACUUUGUACUA 667	Cleavage	1
zma-miR171h-5p	T13_Unigene_BMK.49912	3.0	14.19	miRNA 20 GUACUCGGCUUUGUUAUGGU 1<br>::::.::.::.::::::: :<br>Target 101 CAUGGGCUGAGACAAUACAA 120	Cleavage	1

Table S3 Putative miRNA target of functional encoding gene (Part 1. Key miRNA target of Functional gene at sense strand)

miRNA Acc.	Target Acc.	Expectation (E)	Target Accessibility (UPE)	Alignment	Inhibition	Multiplicity
ath-miR414	T13_Unigene_BMK.48389	1.0	7.324	miRNA 20 CUGCUACUACUACUUCUACU 1<br>::::::::::: ::::::::<br>Target 718 GACGAUGAUGACGAAGAUGA 737	Translation	1
ath-miR414	T19_Unigene_BMK.62787	1.5	7.98	miRNA 21 ACUGCUACUACUACUUCUACU 1<br>:::::::::::. ::::::::<br>Target 2876 UGACGAUGAUGGGGAAGAUGA 2896	Translation	1
ath-miR414	T1_Unigene_BMK.33910	1.5	15.027	miRNA 20 CUGCUACUACUACUUCUACU 1<br>:.:::::::::::::::::<br>Target 1320 UAUGAUGAUGAUGAAGAUGA 1339	Cleavage	1
ath-miR414	T13_Unigene_BMK.13041	1.5	12.181	miRNA 20 CUGCUACUACUACUUCUACU 1<br>:..:::::::.:::::::::<br>Target 140 GGUGAUGAUGGUGAAGAUGA 159	Cleavage	1
ath-miR414	T13_Unigene_BMK.27966	1.5	10.74	miRNA 20 CUGCUACUACUACUUCUACU 1<br>::::: ::::::::.:::::<br>Target 625 GACGACGAUGAUGAGGAUGA 644	Cleavage	1
ath-miR414	T16_Unigene_BMK.22041	2.0	16.895	miRNA 21 ACUGCUACUACUACUUCUACU 1<br>:::.::::::::::: :::::<br>Target 2412 UGAUGAUGAUGAUGACGAUGA 2432	Cleavage	1
ath-miR414	T13_Unigene_BMK.42741	2.0	14.272	miRNA 21 ACUGCUACUACUACUUCUACU 1<br>:::.::::::::::: :::::<br>Target 2726 UGAUGAUGAUGAUGAUGAUGA 2746	Cleavage	1
ath-miR414	T16_Unigene_BMK.19646	2.0	4.233	miRNA 21 ACUGCUACUACUACUUCUACU 1<br>:::.:::::::::::::: ::<br>Target 1096 UGAUGAUGAUGAUGAAGAAGA 1116	Cleavage	1

ath-miR414	T13_Unigene_BMK.34162	2.0	12.72	miRNA 21 ACUGCUACUACUACUUCUACU 1<br>::::::::::::::: ::::.<br>Target 651 UGACGAUGAUGAUGAUGAUGG 671	Cleavage	1
ath-miR414	T16_Unigene_BMK.29836	2.0	4.501	miRNA 20 CUGCUACUACUACUUCUACU 1<br>::.::::::::::: :::::<br>Target 1 GAUGAUGAUGAUGAUGAUGA 20	Cleavage	1
ath-miR414	T19_Unigene_BMK.64323	2.0	13.793	miRNA 20 CUGCUACUACUACUUCUACU 1<br>::.::::::::::: :::::<br>Target 1 GAUGAUGAUGAUGAUGAUGA 20	Cleavage	1
ath-miR414	T13_Unigene_BMK.36273	2.0	15.59	miRNA 21 ACUGCUACUACUACUUCUACU 1<br>:: :: :::::::::::::::<br>Target 1638 UGCCGCUGAUGAUGAAGAUGA 1658	Cleavage	1
ath-miR414	T16_Unigene_BMK.12435	2.0	23.188	miRNA 20 CUGCUACUACUACUUCUACU 1<br>::.:: ::::::::.:::::<br>Target 187 GAUGACGAUGAUGAGGAUGA 206	Cleavage	1
ath-miR5658	T4_Unigene_BMK.47241	1.0	6.609	miRNA 21 AAAGUAGUAGUAGUAGUAGUA 1<br>: :::::::::::::::::::<br>Target 2232 UAUCAUCAUCAUCAUCAUCAU 2252	Cleavage	1
ath-miR5658	T16_Unigene_BMK.89780	1.0	16.244	miRNA 20 AAGUAGUAGUAGUAGUAGUA 1<br>:::::::::::::::::::<br>Target 2 AUCAUCAUCAUCAUCAUCAU 21	Cleavage	1
ath-miR5658	T19_Unigene_BMK.38588	1.0	16.986	miRNA 20 AAGUAGUAGUAGUAGUAGUA 1<br>:::::::::::::::::::<br>Target 774 AUCAUCAUCAUCAUCAUCAU 793	Cleavage	1
ath-miR5658	T16_Unigene_BMK.15038	1.0	4.669	miRNA 20 AAGUAGUAGUAGUAGUAGUA 1<br>::::::: ::::::::::::<br>Target 1114 UUCAUCAACAUCAUCAUCAU 1133	Cleavage	1

ath-miR5658	T10_Unigene_BMK.907	1.5	9.279	miRNA 21 AAAGUAGUAGUAGUAGUAGUA 1<br>:::::::::::::::::::.<br>Target 889 UUUCAUCAUCAUCAUCAUCGA 909	Cleavage	1
ath-miR5658	T16_Unigene_BMK.25895	1.5	12.044	miRNA 21 AAAGUAGUAGUAGUAGUAGUA 1<br>::::::::::::::::: :::<br>Target 92 UUUCAUCAUCAUCAUCAACAU 112	Cleavage	1
ath-miR5658	T16_Unigene_BMK.17592	1.5	11.222	miRNA 20 AAGUAGUAGUAGUAGUAGUA 1<br>:::::::::::::::::.:<br>Target 1301 GUCAUCAUCAUCAUCAUCGU 1320	Cleavage	1
ath-miR5658	T10_Unigene_BMK.9034	1.5	11.928	miRNA 20 AAGUAGUAGUAGUAGUAGUA 1<br>:::::::::::::::::.:<br>Target 5821 AUCAUCAUCAUCAUCAUCGU 5840	Cleavage	1
ath-miR5658	T16_Unigene_BMK.5174	1.5	10.367	miRNA 20 AAGUAGUAGUAGUAGUAGUA 1<br>:::::::::.::.:::::.:<br>Target 622 UUCAUCAUCGUCGUCAUCGU 641	Cleavage	1
ath-miR5658	T16_Unigene_BMK.10132	1.5	13.011	miRNA 20 AAGUAGUAGUAGUAGUAGUA 1<br>::::::: :::.::::::::<br>Target 592 UUCAUCACCAUUAUCAUCAU 611	Cleavage	1
ath-miR5658	T10_Unigene_BMK.81377	1.5	10.088	miRNA 20 AAGUAGUAGUAGUAGUAGUA 1<br>::::::.::::: :::::::<br>Target 494 UUCAUCGUCAUCUUCAUCAU 513	Cleavage	1
ath-miR5658	T16_Unigene_BMK.12539	2.0	24.211	miRNA 21 AAAGUAGUAGUAGUAGUAGUA 1<br>: :::::::::::.::.::::<br>Target 294 UGUCAUCAUCAUCGUCGUCAU 314	Cleavage	1
ath-miR5658	T16_Unigene_BMK.10297	2.0	14.148	miRNA 20 AAGUAGUAGUAGUAGUAGUA 1<br>::::::::::::::::::<br>Target 1416 AACAUCAUCAUCAUCAUCAU 1435	Cleavage	1

ath-miR5658	T4_Unigene_BMK.29725	2.0	24.02	miRNA 20 AAGUAGUAGUAGUAGUAGUA 1<br>::::::::::::::::::<br>Target 1318 AUCAUCAUCAUCAUCAUCAC 1337	Cleavage	1
ath-miR5658	T10_Unigene_BMK.70673	2.0	23.174	miRNA 20 AAGUAGUAGUAGUAGUAGUA 1<br>::.::.:::::::::::::<br>Target 1366 CUCGUCGUCAUCAUCAUCAU 1385	Cleavage	1
ath-miR5658	T16_Unigene_BMK.29578	2.0	6.889	miRNA 20 AAGUAGUAGUAGUAGUAGUA 1<br>::::::::.::.:::::::<br>Target 4987 AUCAUCAUCGUCGUCAUCAU 5006	Cleavage	1
ath-miR5658	T16_Unigene_BMK.26908	2.0	14.672	miRNA 20 AAGUAGUAGUAGUAGUAGUA 1<br>:::.::.:::::.::.::::<br>Target 363 UUCGUCGUCAUCGUCGUCAU 382	Cleavage	1
ath-miR5658	T4_Unigene_BMK.32314	2.0	13.969	miRNA 20 AAGUAGUAGUAGUAGUAGUA 1<br>::::::::: :::::::::<br>Target 1032 AUCAUCAUCACCAUCAUCAU 1051	Translation	1
ath-miR5658	T13_Unigene_BMK.51915	2.0	22.688	miRNA 20 AAGUAGUAGUAGUAGUAGUA 1<br>::::::::::: :::::::<br>Target 821 GUCAUCAUCAUCCUCAUCAU 840	Cleavage	1
ath-miR5658	T16_Unigene_BMK.27441	2.0	13.95	miRNA 20 AAGUAGUAGUAGUAGUAGUA 1<br>::::::::: :::::::::<br>Target 1102 AUCAUCAUCACCAUCAUCAU 1121	Translation	1
ath-miR5658	T16_Unigene_BMK.11012	2.0	5.924	miRNA 20 AAGUAGUAGUAGUAGUAGUA 1<br>:::::: :::::::.::.:<br>Target 418 UUCAUCUUCAUCAUCGUCGU 437	Cleavage	1
ath-miR5658	T13_Unigene_BMK.35056	2.0	18.329	miRNA 20 AAGUAGUAGUAGUAGUAGUA 1<br>: ::::::::::: :::::::<br>Target 2042 UACAUCAUCAUCUUCAUCAU 2061	Cleavage	1

hvu-miR5049f	T19_Unigene_BMK.71641	1.0	15.912	miRNA 21 AGGGAGGCUAGGUAUAAUUAA 1<br>::::::: :::::::::::::<br>Target 2578 UCCCUCCAAUCCAUAUUAAUU 2598	Cleavage	1
hvu-miR5049f	T19_Unigene_BMK.56558	2.0	10.913	miRNA 21 AGGGAGGCUAGGUAUAAUUAA 1<br>:::::.::::::. :::::::<br>Target 43 UCCCUUCGAUCCGAAUUAAUU 63	Cleavage	1
osa-miR1439	T16_Unigene_BMK.26955	1.5	11.548	miRNA 20 UAUGAGUGAGGCAAGGUUUU 1<br>.::::: :::::::::::::<br>Target 2198 GUACUCCCUCCGUUCCAAAA 2217	Cleavage	1
osa-miR1439	T10_Unigene_BMK.6388	1.5	11.725	miRNA 20 UAUGAGUGAGGCAAGGUUUU 1<br>.::::: :::::::::::::<br>Target 426 GUACUCCCUCCGUUCCAAAA 445	Cleavage	1
osa-miR1439	T19_Unigene_BMK.43766	2.0	7.781	miRNA 21 UUAUGAGUGAGGCAAGGUUUU 1<br>:.::::: :::::::::.:::<br>Target 881 AGUACUCUCUCCGUUCCGAAA 901	Cleavage	1
osa-miR1439	T10_Unigene_BMK.6480	2.0	12.132	miRNA 20 UAUGAGUGAGGCAAGGUUUU 1<br>.:::::::::::::: :::<br>Target 2132 GUACUCACUCCGUUCCUAAA 2151	Cleavage	1
osa-miR1439	T4_Unigene_BMK.44207	2.0	23.6	miRNA 20 UAUGAGUGAGGCAAGGUUUU 1<br>:::::: ::.:::::::::.<br>Target 1079 AUACUCCCUUCGUUCCAAAG 1098	Cleavage	1
tae-miR1137a	T16_Unigene_BMK.26955	1.0	12.333	miRNA 20 CUACUGAGUUGAAACAUGAU 1<br>:::::::::::::::::::<br>Target 2220 AAUGACUCAACUUUGUACUA 2239	Cleavage	1
ath-miR399b	T13_Unigene_BMK.48210	1.5	17.257	miRNA 21 GUCCCGUUGAGAGGAAACCGU 1<br>.::::::: :::::::::::.<br>Target 636 UAGGGCAAAUCUCCUUUGGCG 656	Cleavage	2

ath-miR399b	T13_Unigene_BMK.48210	1.5	20.193	miRNA 21 GUCCCGUUGAGAGGAAACCGU 1<br>..:::::: ::::::::::::<br>Target 748 UGGGGCAAAUCUCCUUUGGCA 768	Cleavage	2

Table S3 Putative miRNA target of functional encoding gene (Part2. Key miRNA target of Functional gene at antisense strand**)**

miRNA Acc.	Target Acc.	Expectation (E)	Target Accessibility (UPE)	Alignment	Inhibition	Multiplicity
ath-miR414	T16_Unigene_BMK.5174	1.0	13.783	miRNA 21 ACUGCUACUACUACUUCUACU 1<br>:::::: ::::::::::::::<br>Target 300 UGACGACGAUGAUGAAGAUGA 320	Cleavage	1
ath-miR414	T16_Unigene_BMK.11012	1.0	12.418	miRNA 20 CUGCUACUACUACUUCUACU 1<br>::::: ::::::::::::::<br>Target 833 GACGACGAUGAUGAAGAUGA 852	Cleavage	1
ath-miR414	T10_Unigene_BMK.4545	1.0	9.287	miRNA 20 CUGCUACUACUACUUCUACU 1<br>:::::::: :::::::::::<br>Target 657 GACGAUGAGGAUGAAGAUGA 676	Cleavage	1
ath-miR414	T10_Unigene_BMK.9034	1.5	12.329	miRNA 21 ACUGCUACUACUACUUCUACU 1<br>:::.::::::::::.::.:::<br>Target 558 UGAUGAUGAUGAUGGAGGUGA 578	Cleavage	1
ath-miR414	T10_Unigene_BMK.81377	1.5	8.563	miRNA 21 ACUGCUACUACUACUUCUACU 1<br>:::.:: ::::::::::::::<br>Target 1011 UGAUGAGGAUGAUGAAGAUGA 1031	Cleavage	1
ath-miR414	T19_Unigene_BMK.56736	1.5	14.699	miRNA 20 CUGCUACUACUACUUCUACU 1<br>::.:: ::::::::::::::<br>Target 793 GAUGAGGAUGAUGAAGAUGA 812	Cleavage	1

ath-miR414	T4_Unigene_BMK.47241	2.0	14.875	miRNA 21 ACUGCUACUACUACUUCUACU 1<br>:::.::::::::::: :::::<br>Target 1328 UGAUGAUGAUGAUGAUGAUGA 1348	Cleavage	1
ath-miR414	T19_Unigene_BMK.38588	2.0	14.343	miRNA 21 ACUGCUACUACUACUUCUACU 1<br>:::.::::::::::: :::::<br>Target 2 UGAUGAUGAUGAUGAUGAUGA 22	Cleavage	1
ath-miR414	T16_Unigene_BMK.5881	2.0	17.051	miRNA 21 ACUGCUACUACUACUUCUACU 1<br>:::.:::::::: ::.:::::<br>Target 381 UGAUGAUGAUGAAGAGGAUGA 401	Translation	1
ath-miR414	T10_Unigene_BMK.55727	2.0	13.324	miRNA 20 CUGCUACUACUACUUCUACU 1<br>:: :::::::: ::::::::<br>Target 141 GAAGAUGAUGAAGAAGAUGA 160	Translation	1
ath-miR5658	T19_Unigene_BMK.64323	0.0	13.571	miRNA 20 AAGUAGUAGUAGUAGUAGUA 1<br>::::::::::::::::::::<br>Target 1756 UUCAUCAUCAUCAUCAUCAU 1775	Cleavage	1
ath-miR5658	T16_Unigene_BMK.19646	0.5	2.877	miRNA 20 AAGUAGUAGUAGUAGUAGUA 1<br>::::::::::::::::::.:<br>Target 557 UUCAUCAUCAUCAUCAUCGU 576	Cleavage	1
ath-miR5658	T1_Unigene_BMK.29698	1.0	18.337	miRNA 21 AAAGUAGUAGUAGUAGUAGUA 1<br>::::::::::.::::::::.:<br>Target 453 UUUCAUCAUCGUCAUCAUCGU 473	Cleavage	1
ath-miR5658	T7_Unigene_BMK.44300	1.0	11.807	miRNA 21 AAAGUAGUAGUAGUAGUAGUA 1<br>: :::::::::::::::::::<br>Target 684 UAUCAUCAUCAUCAUCAUCAU 704	Cleavage	1
ath-miR5658	T10_Unigene_BMK.8301	1.0	18.633	miRNA 20 AAGUAGUAGUAGUAGUAGUA 1<br>:::::::::::::::::::<br>Target 1467 AUCAUCAUCAUCAUCAUCAU 1486	Cleavage	1

ath-miR5658	T16_Unigene_BMK.29836	1.0	5.601	miRNA 20 AAGUAGUAGUAGUAGUAGUA 1<br>:::::::::::::::::::<br>Target 7136 GUCAUCAUCAUCAUCAUCAU 7155	Cleavage	1
ath-miR5658	T19_Unigene_BMK.58637	1.5	9.318	miRNA 21 AAAGUAGUAGUAGUAGUAGUA 1<br>::::.:::::::::::::::<br>Target 222 UUUCGUCAUCAUCAUCAUCAA 242	Cleavage	1
ath-miR5658	T4_Unigene_BMK.28658	1.5	15.182	miRNA 21 AAAGUAGUAGUAGUAGUAGUA 1<br>: :.:::::::::::::::::<br>Target 290 UAUUAUCAUCAUCAUCAUCAU 310	Cleavage	1
ath-miR5658	T16_Unigene_BMK.22041	1.5	12.582	miRNA 21 AAAGUAGUAGUAGUAGUAGUA 1<br>: :::::.:::::::::::::<br>Target 421 UAUCAUCGUCAUCAUCAUCAU 441	Cleavage	1
ath-miR5658	T13_Unigene_BMK.34162	1.5	9.969	miRNA 20 AAGUAGUAGUAGUAGUAGUA 1<br>::::::::::::::.::::<br>Target 931 AUCAUCAUCAUCAUCGUCAU 950	Cleavage	1
ath-miR5658	T16_Unigene_BMK.19281	1.5	10.199	miRNA 20 AAGUAGUAGUAGUAGUAGUA 1<br>:::::::::::..::::.::<br>Target 1900 UUCAUCAUCAUUGUCAUUAU 1919	Cleavage	1
ath-miR5658	T19_Unigene_BMK.55475	1.5	14.776	miRNA 20 AAGUAGUAGUAGUAGUAGUA 1<br>::: :::::::::::::.::<br>Target 61 UUCUUCAUCAUCAUCAUUAU 80	Cleavage	1
ath-miR5658	T10_Unigene_BMK.81059	2.0	9.984	miRNA 21 AAAGUAGUAGUAGUAGUAGUA 1<br>:::.:::::::::.::.:.::<br>Target 20 UUUUAUCAUCAUCGUCGUUAU 40	Cleavage	1
ath-miR5658	T4_Unigene_BMK.32314	2.0	7.775	miRNA 20 AAGUAGUAGUAGUAGUAGUA 1<br>::::::::::::::::::<br>Target 3 CUCAUCAUCAUCAUCAUCAC 22	Cleavage	1

ath-miR5658	T19_Unigene_BMK.54201	2.0	13.05	miRNA 20 AAGUAGUAGUAGUAGUAGUA 1<br>::::::::::::::::::<br>Target 144 AGCAUCAUCAUCAUCAUCAU 163	Cleavage	1
ath-miR5658	T10_Unigene_BMK.1973	2.0	14.692	miRNA 21 AAAGUAGUAGUAGUAGUAGUA 1<br>: :.::.::::::::::::::<br>Target 1976 UAUUAUUAUCAUCAUCAUCAU 1996	Cleavage	1
ath-miR5658	T13_Unigene_BMK.42274	2.0	19.615	miRNA 20 AAGUAGUAGUAGUAGUAGUA 1<br>::::::::::::::::::<br>Target 659 GGCAUCAUCAUCAUCAUCAU 678	Cleavage	1
ath-miR5658	T13_Unigene_BMK.28414	2.0	9.1	miRNA 21 AAAGUAGUAGUAGUAGUAGUA 1<br>: ::::::::.::.:::::::<br>Target 526 UGUCAUCAUCGUCGUCAUCAU 546	Cleavage	1
ath-miR5658	T7_Unigene_BMK.52502	2.0	10.863	miRNA 20 AAGUAGUAGUAGUAGUAGUA 1<br>::::::::::::::.::.:<br>Target 264 AUCAUCAUCAUCAUCGUCGU 283	Cleavage	1
ath-miR5658	T4_Unigene_BMK.44197	2.0	11.152	miRNA 20 AAGUAGUAGUAGUAGUAGUA 1<br>::.::.:::::::::::::<br>Target 307 AUCGUCGUCAUCAUCAUCAU 326	Cleavage	1
ath-miR5658	T10_Unigene_BMK.5249	2.0	13.484	miRNA 20 AAGUAGUAGUAGUAGUAGUA 1<br>::::::::.:::::.::::<br>Target 1902 AUCAUCAUCGUCAUCGUCAU 1921	Cleavage	1
ath-miR5658	T10_Unigene_BMK.12425	2.0	11.309	miRNA 21 AAAGUAGUAGUAGUAGUAGUA 1<br>::: ::::::::::::::::<br>Target 94 UUUACUCAUCAUCAUCAUCAU 114	Cleavage	1
ath-miR5658	T1_Unigene_BMK.33910	2.0	7.872	miRNA 20 AAGUAGUAGUAGUAGUAGUA 1<br>::::: :::::::::::::<br>Target 237 GUCAUCUUCAUCAUCAUCAU 256	Cleavage	1

ath-miR5658	T10_Unigene_BMK.48448	2.0	11.308	miRNA 20 AAGUAGUAGUAGUAGUAGUA 1<br>: ::::.:::::::::::.:<br>Target 1427 UGCAUCGUCAUCAUCAUCGU 1446	Cleavage	1
ath-miR5658	T16_Unigene_BMK.16659	2.0	16.06	miRNA 20 AAGUAGUAGUAGUAGUAGUA 1<br>::::::: ::::::::::::<br>Target 1205 UUCAUCA-CAUCAUCAUCAU 1223	Cleavage	1
hvu-miR5049f	T10_Unigene_BMK.75803	0.5	23.871	miRNA 20 GGGAGGCUAGGUAUAAUUAA 1<br>:::::::::::::.::::::<br>Target 1724 CCCUCCGAUCCAUGUUAAUU 1743	Cleavage	1
hvu-miR5049f	T16_Unigene_BMK.27396	1.5	3.041	miRNA 20 GGGAGGCUAGGUAUAAUUAA 1<br>::: :.::::::::::::::<br>Target 3078 CCCACUGAUCCAUAUUAAUU 3097	Cleavage	1
bdi-miR394	T4_Unigene_BMK.41043	0.0	19.287	miRNA 20 CCUCCACCUGUCUUACGGUU 1<br>::::::::::::::::::::<br>Target 2201 GGAGGUGGACAGAAUGCCAA 2220	Cleavage	1
osa-miR1439	T16_Unigene_BMK.72678	1.0	13.937	miRNA 21 UUAUGAGUGAGGCAAGGUUUU 1<br>.:::::: :::::::::::::<br>Target 1967 GAUACUCCCUCCGUUCCAAAA 1987	Cleavage	1
osa-miR1439	T13_Unigene_BMK.28669	1.5	15.162	miRNA 21 UUAUGAGUGAGGCAAGGUUUU 1<br>::::::: :::::::::.:::<br>Target 218 AAUACUCCCUCCGUUCCGAAA 238	Cleavage	1
osa-miR1439	T16_Unigene_BMK.65182	1.5	11.224	miRNA 21 UUAUGAGUGAGGCAAGGUUUU 1<br>..::::: :::::::::::::<br>Target 768 GGUACUCCCUCCGUUCCAAAA 788	Cleavage	1
osa-miR1439	T16_Unigene_BMK.93091	1.5	18.641	miRNA 21 UUAUGAGUGAGGCAAGGUUUU 1<br>..::::: :::::::::::::<br>Target 2352 GGUACUCCCUCCGUUCCAAAA 2372	Cleavage	1

osa-miR1439	T4_Unigene_BMK.31481	1.5	19.422	miRNA 20 UAUGAGUGAGGCAAGGUUUU 1<br>:::::: :::.:::::::::<br>Target 1003 AUACUCCCUCUGUUCCAAAA 1022	Cleavage	1
osa-miR1439	T19_Unigene_BMK.59931	2.0	19.733	miRNA 21 UUAUGAGUGAGGCAAGGUUUU 1<br>: ::::: :::::::::::::<br>Target 2201 ACUACUCCCUCCGUUCCAAAA 2221	Cleavage	1
osa-miR1439	T10_Unigene_BMK.6441	2.0	9.52	miRNA 21 UUAUGAGUGAGGCAAGGUUUU 1<br>::::::: :::: ::::::::<br>Target 3015 AAUACUCCCUCCCUUCCAAAA 3035	Translation	1
osa-miR1439	T19_Unigene_BMK.60601	2.0	7.638	miRNA 20 UAUGAGUGAGGCAAGGUUUU 1<br>:::::: ::::::::::::<br>Target 6949 AUACUCCCUCCGUUCCAAAU 6968	Cleavage	1
tae-miR1137a	T4_Unigene_BMK.31481	0.5	12.423	miRNA 20 CUACUGAGUUGAAACAUGAU 1<br>::::::::::.:::::::::<br>Target 1025 GAUGACUCAAUUUUGUACUA 1044	Cleavage	1
tae-miR1137a	T10_Unigene_BMK.64171	2.0	11.144	miRNA 20 CUACUGAGUUGAAACAUGAU 1<br>:::::::.:::::: :::::<br>Target 633 GAUGACUUAACUUUAUACUA 652	Cleavage	1

Table S4 PCR primers used for Q-PCR amplification of lincRNA

Primer	Forward primer sequence	Reverse primer sequence
**BMK.9590**	5- GGCTTATCCCATCGCTTCCA-3	5-AAGGGAGGATGCGGATGTTC-3
**BMK.31149**	5-CTGCCACAGGTGATGTCCAT-3	5-ACGGCATCCACACAATACGT-3
**BMK.49993**	5-GGAGAAACTTACTGAAGCCGATAG-3	5-GAGGAAGCACTTGATGGTAACCTA-3
**BMK.69095**	5-CAAGCTGGACTCAGGGTGTT-3	5-GCCTCACACTGAACAGGTTTG-3
**BMK.48402**	5-TCTGTACGCGGACATAAGCA-3	5-GAGCGGCAACATACTCCGTA-3
**BMK.48244**	5-GGCTCACGGAAAATTGGCTT-3	5-ACGTGTCCCAGGATAGCATG-3
**BMK.33064**	5-TTGGCTGGTTCGGGATGAAG-3	5-CAGCTGCCAAGATAACACGC-3
**BMK.61316**	5-CGCTTATCCCATCGCTTCCA-3	5-AAGGGAGGATGCGGATGTTC-3
